# Stochastic Expression of the Interferon-β Gene

**DOI:** 10.1371/journal.pbio.1001249

**Published:** 2012-01-24

**Authors:** Mingwei Zhao, Jiangwen Zhang, Hemali Phatnani, Stefanie Scheu, Tom Maniatis

**Affiliations:** 1 Department of Molecular and Cellular Biology, Harvard University, Cambridge, Massachusetts, United States of America; 2 FAS Research Computing, Harvard University, Cambridge, Massachusetts, United States of America; 3 Columbia University College of Physicians and Surgeons, Department of Biochemistry and Molecular Biophysics, New York, New York, United States of America; 4 Institute of Medical Microbiology and Hospital Hygiene, Universität Düsseldorf, Düsseldorf, Germany; Whitehead Institute, United States of America

## Abstract

Virus infection of mammalian cells induces the production of high levels of type I interferons (IFNα and β), cytokines that orchestrate antiviral innate and adaptive immunity. Previous studies have shown that only a fraction of the infected cells produce IFN. However, the mechanisms responsible for this stochastic expression are poorly understood. Here we report an in depth analysis of IFN-expressing and non-expressing mouse cells infected with Sendai virus. Mouse embryonic fibroblasts in which an internal ribosome entry site/yellow fluorescent protein gene was inserted downstream from the endogenous IFNβ gene were used to distinguish between the two cell types, and they were isolated from each other using fluorescence-activated cell sorting methods. Analysis of the separated cells revealed that stochastic IFNβ expression is a consequence of cell-to-cell variability in the levels and/or activities of limiting components at every level of the virus induction process, ranging from viral replication and expression, to the sensing of viral RNA by host factors, to activation of the signaling pathway, to the levels of activated transcription factors. We propose that this highly complex stochastic IFNβ gene expression evolved to optimize both the level and distribution of type I IFNs in response to virus infection.

## Abstract 2

The analysis of stochastic interferon-beta gene expression in virus-infected mammalian cells reveals that the levels of components required for virtually every step in the virus induction pathway are limiting.

## Author Summary

Eukaryotic cells can respond to extracellular signals by triggering the activation of specific genes. Viral infection of mammalian cells, for example, induces a high level of expression of type I interferons (IFNα and β), proteins required for antiviral immunity that protects cells from the infection. Previous studies have shown that the expression of the IFNβ gene is stochastic, and under optimal conditions only a fraction of the infected cells express the IFNβ gene. At present neither the mechanisms nor functions of this interesting phenomenon are well understood. We have addressed this question by analyzing IFN-expressing and non-expressing mouse cells that were infected with the highly transmissible Sendai virus. We show that stochastic IFNβ gene expression is a consequence of cell-to-cell differences in limiting levels and/or activities of virus components at every level of the virus induction process, from viral replication to expression. These differences include the sensing of viral RNA by host factors, the activation of the signaling pathway, and the levels of activated transcription factors. Our findings reveal the complexity of the regulatory mechanisms controlling stochastic IFNβ gene expression. We propose that the stochastic expression of IFN allows for an even distribution of IFN, thus avoiding over-expression of IFN in infected cells.

## Introduction

Eukaryotic cells respond to extracellular signals and environmental stresses by coordinately activating specific sets of genes. Signals from the cell surface or cytoplasm trigger signaling pathways that culminate in the binding of distinct combinations of coordinately activated transcription factors to promoter and enhancer elements that regulate gene expression. A well-characterized example of this is the activation of type I interferon (IFN) gene expression in response to virus infection or double-stranded RNA (dsRNA) treatment [1],[2]. After infection, viral RNA is detected in the cytoplasm by one of two RNA helicases, retinoic acid-inducible gene I (RIG-I) or melanoma differentiation-associated gene 5 (MDA5), which respond to different types of viruses [3]. RIG-I recognizes short dsRNA or panhandle RNA bearing a 5′ triphosphate group [3], and its activity is positively regulated by the ubiquitin E3 ligase tripartite motif 25 (Trim25) [4]. When RIG-I or MDA5 bind to RNA, they form heterodimers, undergo a conformational change, and expose a critical N-terminal caspase-recruiting domain (CARD) [5],[6]. This domain interacts with the CARD domain of the downstream adaptor protein mitochondrial antiviral signaling (MAVS) (also known as IPS-1/Cardif/VISA) on the mitochondrial membrane [7]. The association of RIG-I with MAVS initiates the recruitment of adaptor proteins and leads to the activation of the transcription factors IFN regulatory factors 3 and 7 (IRF3 and IRF7) and NF-κB by the TANK-binding kinase 1 (TBK1) [8]–[10] and IKKα and IKKβ, respectively [7],[11]. Activated IRF3/IRF7 and NF-κB translocate into the nucleus and, along with the transcription factors ATF2/cJun, bind the IFN-β gene enhancer and recruit additional transcription components to form an enhanceosome [12]. This complex signaling and promoter recognition mechanism functions to coordinately activate a specific set of transcription factors that recognize the unique enhancer sequence of the IFNβ gene and thus specifically activate IFN gene expression.

Early in situ hybridization (ISH) studies revealed that induction of IFNβ expression by virus infection or dsRNA treatment in both human and mouse cells is stochastic [13],[14]. That is, only a fraction of the infected cells express IFNβ. This “noisy” expression is not due to genetic variation within the cell population, as multiple subclones of individual cells display the same low percentage of cells expressing IFNβ [14]. In addition, different mouse and human cell lines display different percentages of expressed cells, and the levels of IFNβ gene expression can be increased in low expressing cell lines by fusing them with high expressing lines, or by treating low expressing lines with IFNβ [13],[14]. These studies suggest that stochastic expression of the IFNβ gene is a consequence of cell-to-cell differences in limiting cellular components required for IFN induction, and that one or more of the limiting factors are inducible by IFNβ [13].

Stochastic expression has been observed with a number of other cytokine genes, including *IL-2* [15], *IL-4* [16],[17], *IL-10* [18], *IL-5*, and *IL-13* [19]. In many of these cases, expression is both stochastic and monoallelic. Recent studies of IFNβ gene expression revealed that stochastic expression in human cells is initially monoallelic, and becomes biallelic later in the induction [20],[21]. In one study the stochastic expression of the IFNβ gene was proposed to be a consequence of intrinsic noise due to stochastic enhanceosome assembly [21]. Subsequently, an analysis of human HeLa cells identified a specific set of Alu-repetitive DNA sequences bearing NF-κB binding sites that associate with the IFNβ gene through interchromosomal interactions, and in so doing are thought to increase the local concentration of NF-κB. Initially, only one of the two chromosomes associates with the specialized NF-κB binding sequence, resulting in early monoallelic expression. Secretion of IFN leads to an increased expression of limiting factors (most likely IRF7, which is inducible by IFN), obviating the need for interchromosomal interactions, and leading to the activation of the second IFNβ allele [20]. More recently, heterogeneity in the infecting viruses, rather than cell cycle differences, has been proposed to be the primary source of IFN stochastic expression [22]. Many functions have been proposed for biological noise, ranging from cell fate decisions during development to survival in fluctuating environments [23]. In the case of the IFN genes, neither the mechanisms nor functions of biological noise are well understood.

Here we report a detailed analysis of stochastic IFNβ gene expression in mouse cells. We make use of an IFN-IRES-YFP reporter mouse [24] to perform a detailed analysis of differences between virus-infected cells that either express or do not express IFNβ. Our results reveal a complex picture of stochastic expression of the IFNβ gene, in which the levels of components required for virtually every step in the virus induction pathway are limiting. This includes components required for viral replication and expression, for sensing the presence of viral RNA by the host, and for the virus induction signaling pathway, and the transcription factors required of IFNβ gene expression. Remarkably, in spite of this complexity the percentage of expressing cells remains constant through recloning and cell division, indicating that the stochasm of clonal cells is genetically programmed.

## Results

### Stochastic Expression of Mouse and Human IFNβ Genes

**Figure 1:**
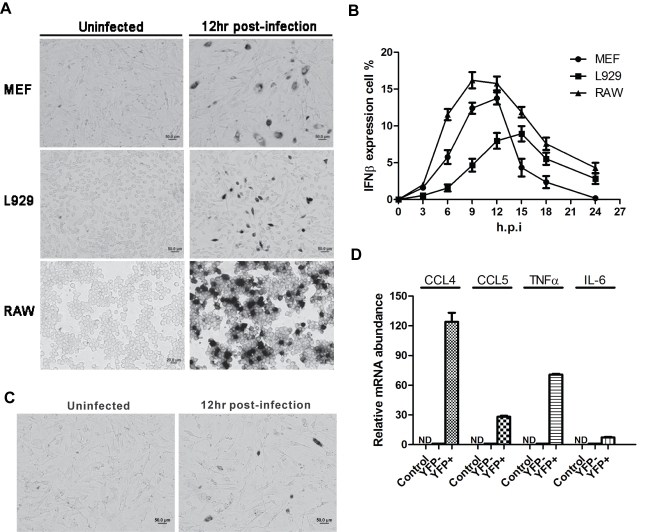
Stochastic IFN and virus-inducible gene expression. (A) Stochastic IFNβ gene expression detected by ISH using a digoxygenin-labeled IFNβ RNA probe. (B) Percentage of IFNβ-producing cells at different times after SeV infection. (C) Mouse IFNα gene expression in primary MEFs detected by ISH using a digoxygenin-labeled IFNα4 probe. (D) qPCR analysis illustrating the expression levels of different virus-inducible genes in sorted IFNβ/YFP MEFs.

Sendai virus (SeV) infection of either mouse or human cells leads to the expression of IFNβ mRNA in only a fraction of the infected cells (Figures 1A, 1B, and S1A), and the percentage of expressing cells differs between different cell lines. The time course of mouse IFNβ expression determined by ISH (Figure 1B) is consistent with that from the quantitative PCR (qPCR) analysis (Figure S1B and S1C). Remarkably, the percentage of cells expressing IFN did not exceed 20%, even at the latest time point (Figure 1B). The absence of IFNβ signal in the majority of cells is not an artifact of hybridization, as β-actin mRNA was detected in all cells (Figure S1D). IFNβ mRNA is specifically detected with an antisense IFNβ RNA probe, while no signal is detected with a sense RNA probe (Figure S1E). In addition, similar percentages of IFNβ-expressing cells were detected by immunofluorescent staining using an IFNβ antibody (Figure S1F), strongly supporting the reproducibility and specificity of the IFNβ ISH.

As mentioned above, enhanceosome assembly and limiting amounts of NF-κB have been proposed to be the primary limiting steps in stochastic expression of the human IFNβ gene [20],[21]. To determine whether this stochastic expression is unique to the IFNβ gene because of the complexity of the IFNβ enhanceosome, or is more general, we examined the expression of the IFNα genes, which are coinduced with IFNβ, but have simple enhancer/promoters, and do not require NF-κB [25],[26]. Using either a mouse IFNα4 or human IFNα8 probe, we found that IFNα genes are also stochastically expressed in both mouse and human cells, respectively (Figures 1C and S1G). Although NF-κB has been shown to be a limiting factor in the activation of the human IFNβ gene [20], it is not required for IFNβ expression in mouse cells [27]. Thus, in spite of this difference both the mouse and human IFNβ genes are stochastically expressed. We also examined other virus-inducible genes, and found that they too are stochastically expressed (see below). Each of these virus-inducible genes requires different levels and combinations of transcription factors, yet they are all stochastic. In all of these cases (mouse and human IFNβ and IFNα and the other virus-inducible genes), the common requirement is the RIG-I virus-inducible signaling pathway. We therefore carried out experiments to determine whether limiting components in this pathway contribute to the observed stochastic expression.

### Separation and Characterization of IFN-Expressing and Non-Expressing Cells

To investigate the mechanism of stochastic IFNβ gene expression, we made use of an IFNβ reporter-knock-in mouse, in which YFP expression allows tracking of IFNβ expression at a single-cell level [24]. Using IFNβ/YFP homozygous mouse embryonic fibroblasts (MEFs) and fluorescence-activated cell sorting (FACS), we obtained pure populations of IFNβ-producing and IFNβ-negative cells upon SeV infection. As expected, IFNβ mRNA is high in the YFP-positive cells, and very low in the YFP-negative cells (Figure S2A). As expected, the IFNα2 and IFNα4 genes are also highly expressed in the YFP-positive cells, and not in the YFP-negative cells (Figure S2A). These observations indicate that replication of the infecting virus and/or components in the RIG-I pathway are the limiting steps in the uninduced cells, rather than intrinsic differences in the IFNβ and α promoters.

We also detected the relative mRNA abundance of other virus-inducible genes in IFNβ-expressing and non-expressing cells. As shown in Figure 1D, transcription levels of all tested inflammatory cytokine or chemokine genes are much higher in IFNβ-producing cells compared to nonproducers. Considering the fact that IFNβ-producing cells account for only 10% of the total cell population, we conclude that expression of all these virus-inducible genes is also stochastic and that these genes are coordinately activated with the type I IFN genes. Activation of these virus-inducible genes is known to require the RIG-I signaling pathway [28]–[31]. Thus, our results indicate that stochastic gene expression is due primarily to limiting components in the signaling pathway and not to gene-to-gene variation in the mechanism of gene activation.

In the case of human cells, stochastic expression of the IFNβ gene is randomly monoallelic early and biallelic late in infection, and the activation of the second IFNβ allele is inducible by IFN [20],[21]. However, the nature of allelic expression of the IFNβ gene has not been addressed in mouse cells. By using IFNβ/YFP heterozygous MEFs, we showed that early after infection (<8 h post-infection [h.p.i.]), IFNβ gene expression was primarily monoallelic, while late in infection (8–16 h.p.i.), the majority of IFNβ-expressing cells were both IFNβ and YFP double-positive cells indicating that, as with human cells, a switch to biallelic expression also occurs in mouse cells (Figure S2B).

Previous studies have shown that the levels of IFNβ gene expression can be increased by priming the cells with IFNβ [13]. Using both mouse and human primary fibroblasts, we showed that IFNβ pretreatment also increases the percentages of IFNβ-expressing cells (Figure S3), indicating that the limiting factor(s) contributing to stochastic IFNβ gene expression are, indeed, inducible by IFNβ. One of these IFN-inducible factors is IRF7 ([20] and see below).

### Viral Replication Is More Efficient in IFNβ-Producing Cells

**Figure 2:**
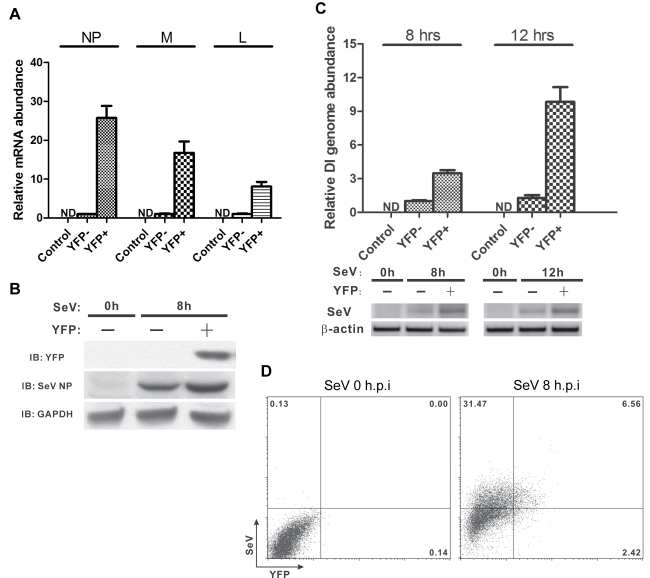
Viral transcription and/or replication are more efficient in IFNβ-producing cells. (A) qPCR analysis illustrating the relative abundance of viral NP, matrix (M), and L polymerase protein (L) mRNA in sorted IFNβ/YFP MEFs. (B) Western blots showing cytoplasmic distribution of SeV NP protein present in IFNβ-producing and nonproducing cells. (C) qPCR analysis illustrating the relative abundance of SeV DI genome (upper panel), and semi-qRT-PCR analysis illustrating the relative abundance of SeV genomic RNA (lower panel) in sorted IFNβ/YFP MEFs. Reverse transcriptase PCR was carried out to detect viral genomic RNA and host cell β-actin mRNA (control) using gene-specific primers. After 35 cycles (SeV genomic RNA) or 26 cycles (β-actin) of amplification, PCR products were run on a 2% agarose gel. (D) Intracellular staining using SeV antibody and FACS analysis were carried out to determine the correlation between SeV infection and IFNβ expression in IFNβ/YFP homozygous MEFs. IB, immunoblot.

To examine the role of the infecting virus in stochastic IFNβ gene expression, we infected primary MEFs with SeV followed by immunofluorescent staining using a SeV antibody. As shown in Figure S4A, most, if not all, of the cells are uniformly infected by SeV, far more than could explain the small percentage of cells expressing IFNβ gene. When we used increasing multiplicities of SeV (as defined by hemagglutination units [HAU]) to infect primary MEFs, we found that the percentage of IFNβ-producing cells increased as the HAU was increased, reaching a maximum of approximately 18% at the peak (Figure S4B). However, as more virus was added (>200 HAU), the percentage of IFNβ-producing cells decreased. Thus, the viral titer is not a limiting factor in the observed stochastic IFNβ gene expression. Next, we determined viral transcript levels in both IFNβ-producing and nonproducing cells. We found that the nucleoprotein (NP), matrix protein, and L polymerase protein mRNA transcripts were present at significantly higher levels in IFNβ-producing cells compared to the nonproducers (Figures 2A and S4C). In addition, higher levels of SeV NP protein were detected in IFNβ-producing cells (Figure 2B).

The RNA helicase RIG-I detects viral genomic RNA and defective interfering (DI) genomes [32],[33]. We therefore examined the levels of viral and DI genomes in both IFNβ-producing and nonproducing cells. As shown in Figure 2C (upper panel), more SeV DI genomes were detected in IFNβ-producing cells compared to IFNβ-nonproducing cells at 8 and 12 h.p.i. Using a primer pair that specifically detects viral genomic RNA, we also detected more viral genomes in IFNβ-producing MEFs 8 and 12 h.p.i. (Figure 2C, lower panel). These results are consistent with the observed viral NP mRNA levels (Figure S4C), and indicate that viral replication is more efficient in the IFN-producing cells. We also investigated the induction activities of total RNA extracted from both IFNβ-producing and nonproducing cells. As shown in Figure S4D, total RNA from IFNβ-producing cells infected for 8 or 12 h induced more IFNβ expression compared to total RNA from IFNβ-nonproducers at the same time points. We conclude that viral mRNA, DI genomes, and viral genomes are present at higher levels in IFNβ-producing cells than in nonproducers. Thus, differences in the efficiency of viral replication/transcription contribute to the stochastic expression of the IFNβ gene.

Previous studies led to the conclusion that the stochastic expression of the IFNβ gene is a feature of the infecting virus, and not of the host cell [22]. To address this possibility, we determined the number of cells that have high levels of viral RNA and produce IFNβ at 8 h.p.i. As shown in Figure 2D, after 8 h of virus infection, approximately 38% SeV-high cells (upper left and upper right) were detected, and about 9% YFP-positive cells (upper right and lower right). Although a higher percentage of IFNβ-expressing cells was observed within the SeV-high cell population (6.56% versus 2.42%), only 17% (6.56% out of 38%) of SeV-high cells produce IFN. Thus, although cell-to-cell differences in viral replication contribute to the stochastic expression of IFN, these differences are not sufficient to explain the extent of stochastic IFN gene expression.

### The RIG-I Signaling Pathway Is Activated and More Potent in IFNβ-Producing Cells

**Figure 3:**
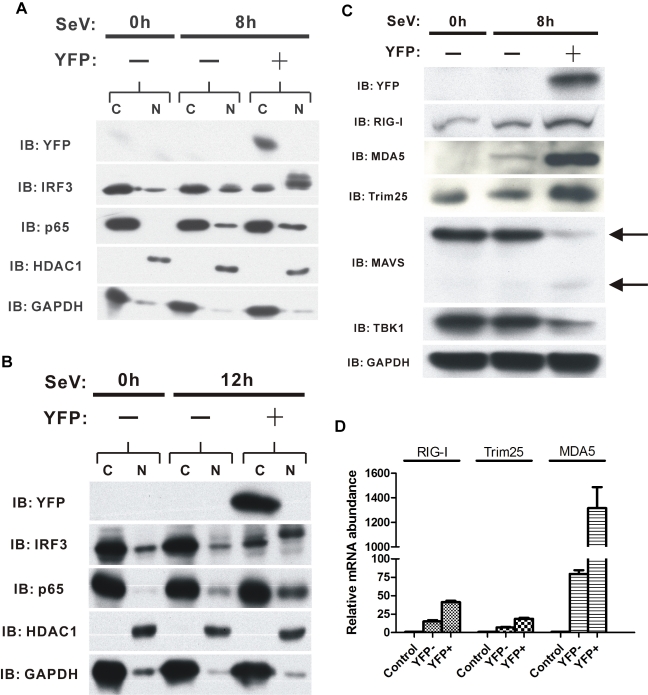
The RIG-I signaling pathway is activated in IFNβ-producing cells. (A and B) Western blots showing cytoplasmic (C) versus nuclear (N) distribution of different factors present in FACS-sorted cells 8 h.p.i. (A) and 12 h.p.i (B). (C) Western blots showing cytoplasmic distribution of signaling pathway proteins present in FACS-sorted cells 8 h.p.i. Arrows indicate MAVS protein. (D) qPCR analysis illustrating the expression levels of RIG-I, Trim25, and MDA5 genes in sorted IFNβ/YFP homozygous MEF cells 8 h.p.i. IB, immunoblot.

To further investigate the mechanism of stochastic IFNβ gene expression, we determined the localization of various components of the signaling pathway required for IFN production using nuclear and cytoplasmic fractions separated from both expressing and non-expressing cells. Consistent with the limiting component hypothesis, we detected phosphorylation and translocation of IRF3 in the YFP-positive cells, but not in the YFP-negative cells (Figure 3A). Previous studies have shown that IRF3, like IRF7, is phosphorylated by the TBK1 kinase, and translocates from the cytoplasm to the nucleus. As both IRF3 and IRF7 are activated via the RIG-I pathway, our results suggest that one or more components of the RIG-I signaling pathway are limiting in the cells that fail to express IFN. A similar result was obtained with sorted cells at 12 h.p.i. (Figure 3B).

In human cells both NF-κB and IRF3/IRF7 are required for virus induction of the IFNβ gene [12],[34]. The human and mouse IFNβ enhancers differ in only two nucleotides out of 45 bases. However, in mouse cells NF-κB is required only for early antiviral activity, when the level of active IRF3 is low, but is not required for maximum levels of IFNβ expression late in induction [27],[35]. Consistent with this finding, we show that only a small fraction of the p65 subunit of NF-κB translocates to the nucleus 8 h.p.i., and little difference is observed in NF-κB localization between the YFP-positive and YFP-negative cells (Figure 3A).

The observation that IRF3 activation and translocation occurs in only a fraction of virus-infected cells suggests that upstream components in the RIG-I signaling pathway differ in IFNβ-producing and nonproducing cells. Western blotting results (Figure 3C) showed that IFNβ-producing cells have higher levels of both RIG-I and MDA5 than the nonproducing population. Trim25, an E3 ligase required for RIG-I activation [4], is also present at a higher level in the IFNβ-producing cells (Figure 3C). The increase in protein levels appears to be a consequence of differential transcription of the tested genes, as mRNA levels of all three genes are higher in IFNβ-producing cells (Figure 3D). We conclude that the IFNβ-producing cells have higher levels of essential RIG-I signaling pathway components than the IFNβ-nonproducing cells. Thus, at least part of the observed stochastic expression is due to limiting RIG-I pathway components in the cells that do not express IFN.

By contrast to the RNA detectors, the protein levels for both MAVS and TBK1, two essential components of the RIG-I signaling pathway [7],[9], were lower in the IFNβ-producing cells (Figure 3C). However, this is likely due to the degradation and/or cleavage of the MAVS protein in infected cells [36]–[38]. The data of Figure 3C suggest that TBK1 is also targeted for degradation during virus infection, consistent with the observation that TBK1 is subject to proteasome-dependent degradation [39]. Thus the turnover of both MAVS and TBK1 may be required for the post-induction turn-off of IFNβ gene expression [38].

### Over-Expression of Individual Components of the RIG-I Signaling Pathway Increases the Percentage of Cells Expressing IFNβ

We have shown that the RIG-I signaling pathway is selectively activated in IFNβ-expressing cells, and this is due only in part to the cell-to-cell differences in virus infection/replication. Our results also suggest that IFNβ-producing cells have a more potent signaling pathway than IFNβ-non-expressing cells. To further explore this possibility, we established a series of L929 stable cell lines that express RIG-I, MDA5, or Trim25 under the control of a tetracycline-inducible promoter (Figure S5A). As shown in Figure S5B and S5C, high levels of exogenous RIG-I only slightly increased the percentage of IFNβ-producing cells. A larger increase was observed with MDA5 and Trim25, but the final percentage in both cases was still under 30%. Thus, these upstream components appear to be among several limiting factors in the cell population.

**Figure 4:**
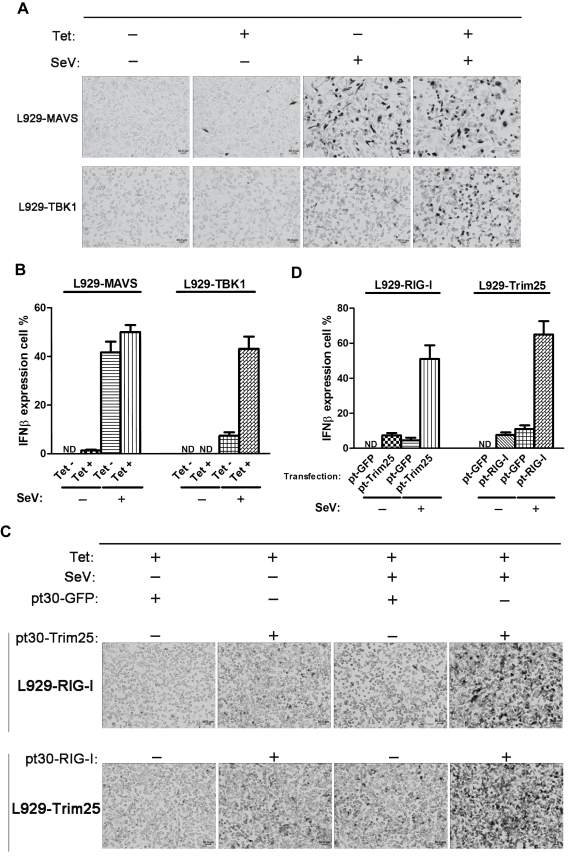
Limiting factors in stochastic IFNβ gene expression. (A) Different L929 stable transfectants were induced by tetracycline (Tet) for 24 h, followed by SeV infection for 9 h. RNA ISH experiments were carried out to detect the IFNβ mRNA. (B and D) Histograms showing the percentage (mean ± standard deviation) of cells expressing IFNβ from three independent ISH experiments. At least 400 cells were blindly counted and scored for each category. (C) L929 stable transfectant was transiently transfected with expression vectors encoding either *GFP* (control), *RIG-I*, or *Trim25*, then stimulated with tetracycline for 24 h. Cells were then infected with SeV for 6 h, followed by RNA ISH to detect IFNβ mRNA. pt, pt-REX-DEST30.

Additional components in the RIG-I signaling pathway were tested using the same approach, and high percentages of IFNβ-producing cells were observed (Figure 4A and 4B). While a large difference between tetracycline-negative and tetracycline-positive cells was observed with the TBK1 line, only a small difference was observed between the corresponding MAVS lines. However, a large difference was observed between the non-transformed and transformed MAVS lines, suggesting that a low level of leaky transcription in the MAVS line is sufficient to dramatically increase the number of IFNβ-expressing cells. These data clearly indicate that both MAVS and TBK1 are limiting components in the RIG-I pathway and therefore contribute significantly to stochastic IFNβ expression.

We have shown that over-expression of RIG-I or Trim25 alone only slightly increases the percentage of IFNβ-producing cells, but it is possible that both must be expressed to achieve maximum levels of IFNβ production. We therefore transfected RIG-I stable transfectants with a Trim25 expression plasmid, and the other way around. The cells were then induced with tetracycline, infected with SeV, and examined for IFNβ mRNA expression. Control experiments using a GFP reporter indicated that under our experimental conditions approximately 70% of cells can be transfected with the second plasmid (Figure S5D). As shown in Figure 4C and 4D, a dramatic increase was observed only 6 h.p.i. when either the RIG-I or Trim25 lines were transfected with Trim25 or RIG-I, respectively. This observation was confirmed by carrying out intracellular staining and flow cytometry experiments using IFNβ/YFP homozygous MEFs (Figure S6). We conclude that the combination of RIG-I and Trim25 is limiting in the RIG-I pathway.

We note that the increase of IFNβ-expressing cells was not observed in uninfected cells, with the only exception being MAVS. Thus, over-expression of these signaling components did not bypass the requirement for signaling pathway activation.

### IRF7 Is a Primary Limiting Factor in Stochastic IFN Gene Expression

**Figure 5:**
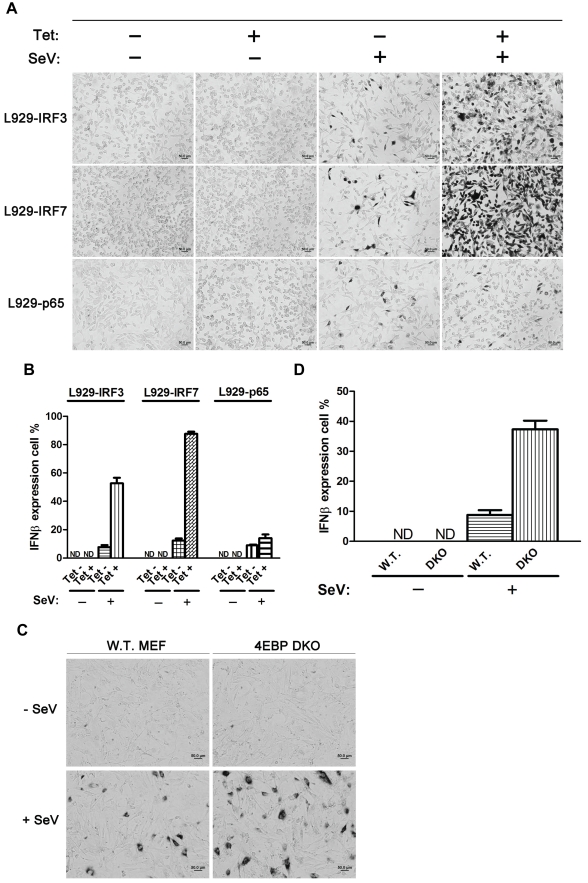
IRF7 is the significant limiting factor in stochastic type I IFN gene expression. (A) Different L929 stable transfectants were induced by tetracycline (Tet) for 24 h, followed by SeV infection for 9 h. RNA ISH experiments were carried out to detect IFNβ mRNA. (B and D) Histograms showing the percentage (mean ± standard deviation) of cells expressing IFNβ from three independent ISH experiments. At least 400 cells were blindly counted and scored for each category. (C) RNA ISH experiments were carried out to detect IFNβ mRNA in wild-type (W.T.) or 4E-BP double-knockout (DKO) MEFs infected by SeV for 9 h.

Expression of the IFNβ gene requires an active RIG-I signaling pathway and assembly of the enhanceosome complex on the IFNβ promoter. To investigate whether individual enhanceosome components are limiting factors, we established a series of tetracycline-inducible L929 stable lines that express IRF3, IRF7, or p65 genes. Figure 5A and 5B show that, without tetracycline induction, only 10%–15% of the cells produce detectable levels of IFNβ mRNA in response to virus infection. Remarkably, the percentage of IFNβ-producing cells upon SeV infection increased to 85% when IRF7 expression was induced by tetracycline in every cell (Figure S7A). A smaller increase (55%) was observed when IRF3 was over-expressed, whereas increasing the concentration of NF-κB had little effect, consistent with the data in Figure 3A, and previously published studies [27]. Interestingly, IRF7 over-expression also significantly increased the percentage of IFNα-producing cells after virus infection (Figure S7B and S7C). It is known that IRF7 is required for maximum induction of type I IFN genes [25], and its basal protein level is very low in most cell types except for plasmacytoid dendritic cells [26],[40]. We conclude that IRF7 is a critical limiting factor that is a major contributor to stochastic expression of mouse IFNα and β genes. This conclusion is also supported by our ISH results from 4E-BP1/4E-BP2 double-knockout MEFs (Figure 5C and 5D). Previous studies have indentified 4E-BPs as negative regulators of type I IFN production via translational repression of IRF7 mRNA [41]. As shown in Figure 5C and 5D, we observed a 4-fold increase of the percentage of IFNβ-expressing cells in 4E-BP1/4E-BP2 double-knockout MEFs compared to wild-type MEFs, consistent with the conclusion that a limiting amount of IRF7 is a major contributor to the stochastic expression of IFNβ.

We also found that type I IFN induction was exceptionally high, with much faster kinetics in cells expressing exogenous IRF7 than in control cells (Figure S7D). In the absence of tetracycline induction, low levels of IFNβ, IFNα4, and IFNα2 mRNA were first detected 6 h, 9 h, and 12 h.p.i., respectively. When the cells were treated with tetracycline, the kinetics of IFN gene transcription changed significantly. IFNβ, IFNα4, and IFNα2 transcripts could be detected as early as 4 h after virus infection. Even at 24 h.p.i., steady and robust transcription of these genes could still be detected. These observations are consistent with a model in which IRF3 is normally activated early for IFN gene induction. Later, higher levels of IRF7 are produced by IFN and are required for both IFNβ and IFNα gene expression, but IRF7 is rapidly turned over, leading to the cessation of both IFNβ and IFNα gene expression [1],[25],[26]. By contrast, in the presence of excess IRF7 in the tetracyline-activated cells, both IFNβ and IFNα are activated earlier, and continue to be expressed because of the continuous presence of IRF7.

### IRF7 Positively Regulates the RIG-I Signaling Pathway

We have shown that over-expression of IRF7 or both RIG-I and Trim25 almost completely eliminates stochastic IFNβ expression (Figures 4C, 4D, and 5). To investigate the connection between these observations, we carried out microarray analysis to compare genome-wide expression profiles of L929-IRF7 stable transfectants treated with or without tetracycline. Interestingly, upon IRF7 over-expression, only two up-regulated signaling pathways were identified from the KEGG Pathway Database, and the RIG-I-like receptor signaling pathway is the most up-regulated (*p* = 3.6E-06) (Figure S8A and S8B) [42]. We did not identify signaling pathways that were similarly enriched among the down-regulated genes. Using qPCR, we confirmed that the mRNA levels of both RIG-I and Trim25 were higher in IRF7 over-expressing cells (Figure S8C). Considering the low basal expression level of IRF7, we conclude that a high level of IRF7 protein increases the percentage of IFNβ-expressing cells not only by increasing its own abundance, but also by up-regulating the RIG-I signaling pathway to increase the potency of activation of the IFNβ gene.

### Stochastic Expression of IFNβ Induced by dsRNA—poly I∶C Is Due to Limiting Amounts of MDA5 and IRF7

**Figure 6:**
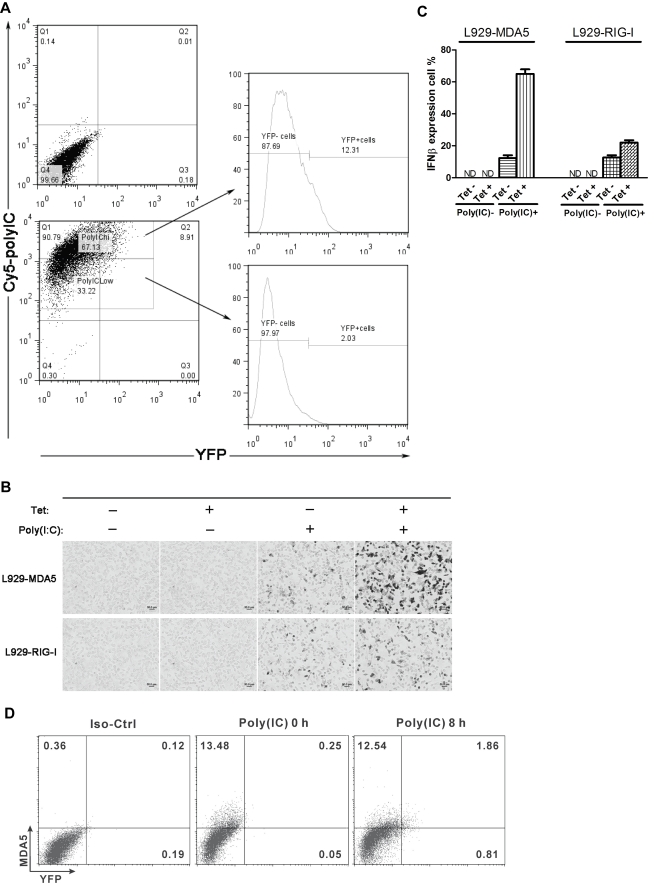
Poly I∶C–induced stochastic IFNβ expression depends on the amounts of poly I∶C and MDA5. (A) IFNβ/YFP homozygous MEF cells were electroporated with Cy5-labeled poly I∶C, and FACS analysis was carried out 8 h after the electroporation to assay the strength of Cy5 and YFP. The top left panel shows untransfected MEF cells, and the bottom left panel shows the electroporated MEF cells. As indicated by arrows, the two panels to the right represent the “poly I∶C high” and “poly I∶C low” populations, respectively. Data shown are representative of at least three independent experiments. Numbers represent relative percentages. (B) L929-MDA5 or L929-RIG-I stable transfectants were stimulated with tetracycline (Tet) for 24 h followed by transient transfection with poly I∶C. 6 h after transfection, cells were fixed, followed by RNA ISH to detect IFNβ mRNA. (C) Bar plots representing the percentage (mean ± standard deviation) of cells expressing IFNβ from three independent ISH experiments performed as in (B). At least 400 cells were blindly counted and scored for each category. (D) IFNβ/YFP primary MEFs were fixed 8 h after poly I∶C stimulation. Intracellular staining using MDA5 antibody and FACS analysis were carried out to assay the correlation between the expression levels of IFNβ and MDA5. Data shown are representative of at least three independent experiments. Numbers represent relative percentages. Iso-Ctrl, isotype control.

IFNβ gene expression can also be induced by transfection of the synthetic dsRNA polyriboinosinic polyribocytidylic acid (poly I∶C), and this induction occurs mainly through the MDA5 signaling pathway [43]. Early studies revealed that induction of IFNβ expression by dsRNA treatment is also stochastic [13],[14]. We therefore asked whether stochastic IFNβ gene expression induced by dsRNA is due to cell-to-cell variation in the levels of MDA5 and IRF7. Using FACS analysis, we found that poly I∶C–induced IFNβ expression is also stochastic (Figure 6A). When IFNβ/YFP homozygous MEFs were electroporated with Cy5-labeled poly I∶C, only 9% of the cells produced IFNβ as detected by the presence of YFP. However, the electroporation efficiency was over 99% (Figure 6A, left panel). Interestingly, based on the Cy5 intensity, there were two populations of cells, which contained different amounts of poly I∶C. When we gated these two populations out as “poly I∶C–high” and “poly I∶C–low”, we observed that the “poly I∶C–high” population included more cells producing IFNβ (Figure 6A, right panel), indicating that the amount of inducer does affect the extent of stochastic IFNβ expression. However, only a small percentage of “poly I∶C–high” cells expressed the IFNβ gene, clearly indicating that other limiting factor(s) dominate the stochastic IFNβ expression induced by poly I∶C transfection. We therefore carried out experiments to identify these limiting components.

L929-MDA5 and L929-RIG-I stable transfectants were transfected with poly I∶C followed by ISH to detect IFNβ expression. As shown in Figure 6B and 6C, over-expression of RIG-I only slightly increased the percentage of IFNβ-producing cells. By contrast over-expression of MDA5, the major cytoplasmic receptor for poly I∶C, led to a substantial increase in the percentage of IFNβ-producing cells (from 15% to 65%). Considering that the transfection efficiency is approximately 75% (data not shown), over-expression of MDA5 basically eliminates stochastic expression of the IFNβ gene in response to poly I∶C transfection. Furthermore, the results of the flow cytometry experiment also supported this conclusion. As shown in Figure 6D, after 8 h of poly I∶C stimulation, we observed approximately 2.6% YFP-positive cells. Within this population, about 70% of the YFP-positive cells had higher levels of MDA5 protein (1.86% out of 2.67%). We note that the percentage of YFP-positive cells is much lower than that observed with virus infection (Figures 2D and S6).

Over-expression of IRF3 or IRF7 also increased the percentage of IFNβ-producing cells in response to poly I∶C (Figure S9A and S9B). As shown in Figure S8, over-expression of the IRF7 gene up-regulates MDA5 gene expression. Considering its low basal expression level, IRF7 is also an important limiting factor in stochastic IFNβ expression induced by poly I∶C transfection. Taken together, these data show that poly I∶C–induced stochastic IFNβ expression depends on the abundance of both poly I∶C and signaling pathway protein MDA5 as well as IRF3/IRF7, which is similar to what was found in the case of virus infection.

### Variation in the Levels of RIG-I Signaling Pathway Components

**Figure 7:**
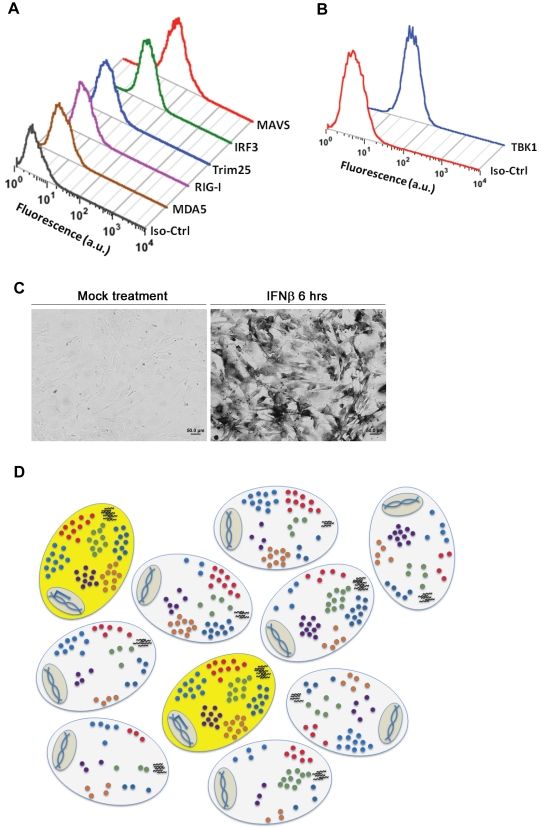
Endogenous variation in the concentrations of components of the RIG-I signaling pathway. (A and B) Protein distributions in untreated primary MEFs determined by flow cytometry. a.u., arbitrary units. (C) Mouse ISG15 gene expression in MEFs 6 h after IFNβ treatment, detected by ISH using a digoxigenin-labeled ISG15 probe. (D) A model depicting stochastic IFN gene expression. There is a population of ten cells with varying numbers of limiting factors in each cell. Each small, colored circle represents one of the limiting factors, and six limiting factors are shown. Short black lines represent viral inducer. Only two cells in the population have enough of the viral inducer and all six factors to trigger transcription of the IFNβ gene. Iso-Ctrl, isotype control.

We also asked whether the concentrations of proteins regulating IFNβ expression are sufficiently different from cell to cell to account for the stochastic IFNβ expression. Using flow cytometry, we measured the distributions of six components in the RIG-I signaling pathway for which specific antibodies are available. As shown in Figure 7A and 7B, all six proteins were log-normally distributed across the population. Quantitative immunofluorescence data for individual components show similar distributions of each factor at the single-cell level (Figure S10). Combined with our previous data, these observations suggest that naturally occurring differences in the protein levels of signaling pathway components are the primary cause of cell-to-cell variability in IFNβ expression upon virus infection.

### IFN-Inducible Antiviral Genes Are Not Stochastically Expressed

When IFN is secreted from virus-infected cells in vivo, it binds to type I IFN receptors on surrounding cells and activates a large set of genes encoding antiviral proteins (interferon-stimulated genes [ISGs]) via the Jak/STAT signal transduction pathway. We therefore carried out experiments to determine whether the induction of antiviral ISGs is also stochastic. As shown in Figure 7C, ISG15 is expressed in all cells upon treatment with IFNβ. Thus, when IFN is secreted, all of the surrounding cells produce antiviral proteins. This result is also consistent with previous observations showing that the antiviral response induced by IFN is a robust feature common to all cells, and is independent of the stochastic expression of IFN receptor IFNAR [44].

## Discussion

Regulation of type I IFN production is essential for the innate immune response to viral infections [45],[46]. However, high levels of IFNβ can be toxic [47],[48]. Thus, IFNβ production must be tightly regulated. This regulation appears to be both temporal and stochastic. Type I IFN genes are tightly repressed prior to virus infection, activated upon infection, and then rapidly turned off several hours later (Figure S1B and S1C). Previous studies of several cytokine genes suggest that this stochastic gene expression provides an additional mechanism of regulation whereby optimal levels of cytokine production are determined by the frequency of expressing cells rather than by protein levels per cell [18],[19],[49]. Thus, it is possible that stochastic expression is a primary mechanism for controlling the optimal level of IFNβ production in vivo. In particular, we have shown that while IFN production is stochastic, the activation of the antiviral gene program by secreted IFN is not. Thus, stochastic expression of IFN would allow the regional distribution of the cytokine and activation of the surrounding cells, without producing toxic levels of IFN.

Previous studies have implicated as limiting steps enhanceosome assembly [20],[21] and the assembly of an interchromosomal transcriptional hub formed through interactions between Alu elements bearing NF-κB sites [20]. More recently, the infecting virus, rather than intrinsic properties of the infected cell, has been implicated in this stochasm [22]. The data presented here reveal a far more complex mechanism in which cell-to-cell variations in limiting components required to support viral replication, to detect and signal the presence of viral RNA, and to activate transcription factors all contribute to the observed stochastic expression (Figure 7D). It seems likely that the key limiting factor varies between cell types, cell lines, and organisms.

The earliest step in the virus induction signaling pathway is entry of virus or dsRNA into the cell. We have shown that both inducers elicit stochastic expression, but in neither case is this due to limiting inducer (Figures S4B and 6A). We showed that both IFNβ-producing and nonproducing cells were infected by SeV (Figure 2B). However, the IFNβ-producing cells contained significantly higher levels of the products of viral replication and transcription. Thus, it appears that there are cell-to-cell differences in the ability to support efficient viral replication, and these differences influence the probability of IFNβ gene expression. Presumably, high levels of RNA inducer in the IFN-producing cells overcome limiting amounts of RIG-I or MDA5. However, differences in viral replication alone cannot explain the observed stochasm in IFNβ production. A previous study, using a cell line transfected with an IFNβ-GFP reporter, concluded that stochastic IFNβ expression is due entirely to heterogeneity in the infecting virus [22]. However, in that study the IFNβ-GFP cell line was preselected to minimize stochastic expression of the reporter. In addition, that study involved a stably transfected gene, while the present study made use of the endogenous gene. The results presented here strongly indicate that heterogeneity of both the virus and host cells together are responsible for the stochastic expression of IFNβ.

We have identified multiple limiting steps in the activation of IFNβ gene expression, ranging from initial steps in virus infection and replication, to the signaling pathway, to the activation and binding of transcriptional activator proteins to the IFNβ promoter. For example, over-expression of individual components in the RIG-I signaling pathway increases the percentage of IFN-expressing cells. The largest increase was observed with IRF7, which lies at the endpoint of the RIG-I pathway, and also positively controls the expression of components in the RIG-I signaling pathway. Taken together, these data are consistent with a model in which the probability of expression of the IFNβ gene in individual cells depends primarily on the activation of the RIG-I signaling pathway and the presence of sufficient numbers of IRF7 molecules to activate transcription (Figure 7D). This conclusion is consistent with the observation that both *IFN*β and *IFN*α are stochastically expressed in response to virus infection (Figure 1A and 1C). The expression of both genes requires activation of the RIG-I pathway and active IRF7 [50].

We find that limiting amounts of other RIG-I pathway components also contribute to stochastic expression of the IFNβ gene, as we observed higher levels of RIG-I/Trim25 and MDA5 mRNA and protein levels in the IFNβ-producing cells than in the nonproducers (Figure 3). In addition, over-expression of RIG-I and Trim25 together leads to a dramatic increase in the percentage of cells that express IFNβ (Figure 4C and 4D). Similar results were obtained with high levels of expression of the RIG-I signaling components MAVS and TBK1 and the transcription factors IRF3 and IRF7 (Figures 4A, 4B, and 5). Thus, it appears that many, if not all, of the components in the RIG-I signaling pathway, from the sensors of viral RNA to the essential transcription factors, can be limiting components in the virus induction pathway.

The largest increase in the percentage of IFN-producing cells was observed when IRF7 was over-expressed. IRF7 is the master regulator of type I IFN gene expression [25], and is present at low levels in all cell types except plasmacytoid dendritic cells, where it is constitutively abundant [26],[40]. Our over-expression experiments show that high levels of IRF7 promote the transcription of type I IFN genes (Figure S7D), and essentially eliminate the stochastic expression of both the IFNβ and α genes (Figures 5 and S7). In a previous study in human cells, both NF-κB and IRF7 over-expression was shown to partially suppress stochastic IFNβ expression [20]. Our results are consistent with this observation. However, there are two differences. First, based, at least in part, on the lack of requirement of NF-κB in murine cells, we observed a relatively small effect of increasing NF-κB expression. Second, we saw a greater effect of IRF7 expression in murine cells than was observed in human cells. Over-expression of IRF7 in L929 cells almost completely eliminated stochastic expression of both IFNβ and α genes, while in human HeLa cells high levels of IRF7 increase the percentage of IFNβ-producing cells to almost 55% [20]. Deleting the IRF7 translational repressors, 4E-BPs, also increased the IFNβ-expressing MEFs by 4-fold (Figure 5C and 5D). We also showed that the RIG-I signaling pathway, and in particular RIG-I and Trim25, are up-regulated in IRF7 over-expressing cells (Figure S8). We conclude that limiting amounts of active IRF7 appear to be overcome by two mechanisms: positive auto-regulation of IRF7 expression, and IRF7-dependent up-regulation of the RIG-I signaling pathway.

We note that in addition to IFNβ, several other virus-inducible genes, including TNFα, IL-6, CCL4, and CCL5, are highly expressed in the IFNβ-producing cells compared to nonproducers, suggesting that many, if not all, of the virus-inducible genes are stochastically expressed. The common feature of the activation of all of these genes is that they all require the RIG-I signaling pathway [28]–[31]. Thus, we conclude that stochastic gene expression is primarily due to limiting components in the signaling pathway but not gene-to-gene variation in the mechanism of gene activation.

We showed that although the IFNβ gene is stochastically expressed upon virus infection, the antiviral ISGs, e.g., ISG15, were equally induced in all cells (Figure 7C). However, we note that RIG-I, Trim25, and MDA5, which are also antiviral ISGs, are highly expressed in IFNβ-producing cells compared to nonproducing cells (Figure 3C and 3D). We believe that the differences we observed here reflect naturally occurring cell-to-cell variability in the levels of expression of these genes prior to virus infection, and that this variability is the primary source of stochastic IFNβ gene expression. However, at later times after virus infection, we expect that the differences in the mRNA or protein levels of these genes between the YFP-positive and YFP-negative populations will be much smaller compared to those at earlier stages (8 h.p.i.). As shown in Figure S11A and S11B, our qPCR data and Western blot data support this expectation. The IFNβ gene is also stochastically expressed in IFNAR-deficient MEFs, which suggests that the IFNAR levels or an IFNβ feedback loop are not major factors responsible for stochastic IFNβ gene expression (Figure S11C). We further measured the distributions of six components in the RIG-I signaling pathway. As shown in Figures 7A, 7B, and S10, all six proteins were log-normally distributed across the cell population, an observation that is consistent with data on other proteins [51],[52]. Thus, naturally occurring differences in the protein levels and activities of individual signaling pathway components and transcription factors account for stochastic IFNβ expression induced by both poly I∶C induction and virus infection.

Previous studies have shown that naturally occurring differences in the levels of proteins in the apoptotic signaling pathway are the primary reasons for cell-to-cell variability in the probability of cell death [52]. Thus, the results presented here not only reveal the complexity of the regulatory mechanisms controlling stochastic IFNβ gene expression, but also suggest a general mechanism used in different biological processes to establish and control stochastic gene expression. A remarkable feature of stochastic expression is that it appears to be an intrinsic property of different clonal populations of cells. For example, if a particular cell line displays a certain percentage of activated cells, that percentage differs from other cell lines, and is retained when the cells are recloned [14]. Thus, the extent of stochasm appears to be a genetic and epigenetic feature of clonal cell populations.

## Materials and Methods

### Cells, Reagents, and Plasmids

All cell lines, including L929, RAW 264.7, MG63, and 293T, were from the American Type Culture Center; primary MEFs were isolated using standard protocols from IFNβ/YFP mice [24]. Primary human foreskin fibroblast cells were purchased from PromoCell. All cells were cultured in DMEM (Gibco) supplemented with 10% FBS (Gibco) in a 5% CO_2_ incubator. Cycloheximide was purchased from Sigma-Aldrich. Human and mouse recombinant IFN proteins were purchased from PBL Interferonsource. Brefeldin A solution was purchased from eBioscience. Poly I∶C was purchased from InvivoGen. Cy5-labeled poly I∶C was generated using Label IT Nucleic Acid Labeling Kit (Mirus). The different expression constructs were generated by cloning the coding sequences of each gene by PCR and inserting them into the vector pt-REX-DEST30, which has the tetracycline-inducible promoter (Invitrogen).

### Virus Infection and Poly I∶C Transfection

Concentrated SeV stock (Cantell strain, Charles River Lab) was added to cultured cells at a concentration of 200 HAU/ml and incubated for the times indicated. Poly I∶C transfection was carried out using either lipofectamine2000 (Invitrogen) or electroporation using Amaxa MEF2 Nucleofector Kit (Lonza).

### RNA Preparation and PCR

Total RNA was extracted with Trizol reagent (Invitrogen). Real-time quantitative reverse transcription PCR (qRT-PCR) was conducted according to standard protocols.

### Antibodies and Western Blot

Antibody against YFP was from Chemicon (Millipore) or Abcam. RIG-I, MAVS, and GAPDH antibodies were from Cell Signaling. Antibodies against p65, HDAC1, and Trim25 were from Santa Cruz Biotechnology. MDA5 and TBK1 antibodies were from Abcam and Imgenex, respectively. IFNβ antibody used for FACS was from Millipore. SeV antibodies were kindly provided by Dr. Atsushi Kato (National Institute of Infectious Diseases, Japan). Nuclear/cytosol fractionation was performed using Nuclear/Cytosol Fractionation Kit (BioVision). Western blots were carried out using standard protocols.

### In Situ Hybridization

Antisense RNA probes recognizing mouse IFNβ or β-actin were synthesized using T7 or SP6 polymerase and digoxigenin-labeled nucleotides (Roche Applied Science). Cells were cultured on poly-D-lysine-coated 24-well plates (Fisher) and either mock- or virus-infected for the times indicated. Cells were then washed twice with PBS and fixed with 4% paraformaldehyde. Hybridization, washes, and staining were carried out as previously described [53].

### Flow Cytometry

MEF cells were fixed with IC Fixation Buffer and permeabilized with Permeabilization Buffer (both from eBioscience). After incubation with appropriate antibodies, flow cytometry was done with a FACSCalibur, and data were analyzed with CellQuest software (both from Becton Dickinson).

### Microarray Analysis and KEGG Pathway Enrichment Analysis

Total RNA from untreated and tetracycline-induced L929-IRF7 cells were prepared using Trizol reagent (Invitrogen) followed by purification using MEGAclear (Ambion). Biotinylated RNA probes were synthesized by two rounds of amplification using the MessageAmp II aRNA Amplification kit (Ambion). The probes were hybridized with Affymetrix Mouse Genome 430A_2.0 array chips. Affymetrix DAT files were processed using the Affymetrix Gene Chip Operating System to create CEL files. Normalized expression values were analyzed with the Bioconductor Limma package, an approach for implementing empirical Bayes linear modeling [42]. For all comparison tests, genes with an absolute fold change in transcript level exceeding 1.5 and *p*<0.05 were selected for further analyses. The likelihood of overrepresentation of KEGG signaling pathways in the up- or down-regulated gene list relative to a background of all array genes was calculated by Fisher's exact test for statistical analysis.

## Supporting Information

Figure S1 **Stochastic expression of IFNβ gene upon virus infection.** (A) Stochastic expression of human IFNβ gene in primary foreskin fibroblast cells, MG63 cells, and 293T cells 6 h.p.i., detected by ISH using a digoxygenin-labeled antisense RNA IFNβ probe. Numbers on the right indicate the percentages of IFNβ-expressing cells in each cell type. (B and C) Kinetics of IFNβ expression in primary MEFs (B) or L929 cells (C) assayed by qRT-PCR. IFNβ mRNA can be detected as early as 6 h.p.i., and maximum levels are observed at 9 and 12 h.p.i. in MEFs and L929 cells, respectively. (D) β-actin mRNA in L929 cells was detected by ISH using either digoxygenin-labeled sense or antisense RNA probe. (E) MEF cells or human MG63 cells were infected by SeV for 9 h or 6 h, respectively. ISH was carried out to detect the IFNβ-expressing cells using an IFNβ sense or antisense probe. (F) Human MG63 cells were infected by SeV for 9 h. IFNβ protein was detected by immunocytochemistry using IFNβ antibody. Similar percentages of IFNβ-expressing cell were detected by either ISH or immunocytochemistry. (G) Human IFNα8 mRNA in Namalwa cells was detected by ISH using digoxygenin-labeled probe.
(TIF)

Figure S2 **IFN expression in sorted MEFs and allelic expression of IFNβ gene.** (A) qRT-PCR analysis illustrating the expression levels of IFN genes in sorted IFNβ/YFP primary MEF cells. (B) IFNβ/YFP heterozygous MEFs (upper panel) and homozygous MEFs (lower panel) were infected by SeV for variable times with the presence of Brefeldin A (BFA)—which inhibits transport of proteins from endoplasmic reticulum to Golgi—in the last 4 h. Cells were fixed and stained for intracellular IFNβ and YFP. If IFNβ gene is monoallelically expressed, heterozygous MEF cells should have similar percentages of IFNβ-positive population (from IFNβ allele) and IFNβ/YFP double-positive population (from IFNβ-IRES-YFP allele). If IFNβ gene is biallelically expressed, all, or at least most, of IFNβ-expressing heterozygous cells should be both IFNβ- and YFP-positive. Heterozygous MEF FACS analysis (upper panel) showed a similar percentage of IFNβ-positive population (upper left panel, 5.24%) and IFNβ/YFP double-positive population (upper right panel, 6.30%) at 8 h.p.i., suggesting that the IFNβ gene expression was predominantly monoallelic before 8 h.p.i. During the time periods 8–12 h.p.i. and 12–16 h.p.i., the majority of IFNβ-expressing cells were IFNβ/YFP double-positive (upper right panel, 11.01%, and upper right panel, 3.90%, respectively), indicating that at late infection, IFNβ gene expression was biallelic. As control, shown in the lower panel, IFNβ-expressing homozygous MEF cells had almost no IFNβ single-positive population at any given time point. Data shown are representative of at least three independent experiments. Numbers represent relative percentages.
(TIF)

Figure S3 **Priming of cells increases the percentage of IFNβ-expressing cells.** (A) Primary MEFs were primed with 250 U/ml IFNβ or 250 U/ml IFNβ plus 50 µg/ml cycloheximide (CHX) for 6 h, infected by SeV, and subjected to ISH using digoxygenin-labeled IFNβ RNA probe. (B) Histogram showing the percentage (mean ± standard deviation) of cells expressing IFNβ from three independent ISH experiments as in (A). (C) Human foreskin fibroblasts were primed with 250 U/ml IFNβ or 250 U/ml IFNβ plus 50 µg/ml cycloheximide for 6 h, infected by SeV, and subjected to ISH using digoxygenin-labeled IFNβ RNA probe. (D) Histogram showing the percentage (mean ± standard deviation) of cells expressing IFNβ from three independent ISH experiments as in (C).
(TIF)

Figure S4 **Viral titer is not a limiting factor.** (A) MEF cells were infected by SeV. Cells were fixed and stained for SeV using SeV antibody. Blue color shows DAPI staining (nucleus) and green color shows SeV signal. Most, if not all, cells are uniformly exposed to SeV. Scale bar, 20 µm. (B) Percentages (mean ± standard deviation) of IFNβ-producing primary MEF cells infected by increasing amounts of SeV. At least 400 cells were counted and scored blindly for each category. In all of the experiments described in this study, we used 100–200 HAU/ml of SeV as the infecting dose. (C) qRT-PCR analysis illustrating the relative abundance of viral NP mRNA in sorted IFNβ/YFP primary MEF populations 8 or 12 h.p.i. (D) L929 cells were transfected with total RNAs either from IFNβ-producing or IFNβ-nonproducing MEF cells sorted after being virus-infected for 8 h or 12 h. Then total RNAs were extracted from these L929 cells 8 h after transfection, and qRT-PCR experiments were carried out to detect relative abundance of IFNβ mRNA in these transfected cells.
(TIF)

Figure S5 **Over-expression of RIG-I, MDA5, or Trim25 increases the percentage of IFNβ-expressing cells.** Different tetracycline-inducible L929 stable transfectants were generated. In the absence of tetracycline, expression of exogenous copies of these genes is tightly repressed in the stable transfectants, but upon the addition of 1 µg/ml tetracycline, the stably incorporated genes are expressed at a high level. (A) Western blots showing the tetracycline-inducible expression levels of different proteins. All genes were flag tagged and proteins were detected using Flag antibody. (B) L929-RIG-I, L929-MDA5, and L929-Trim25 stable transfectants were induced by tetracycline for 24 h, followed by virus infection for 9 h. RNA ISH experiments were carried out to detect the IFNβ mRNA. (C) Histogram showing the percentage (mean ± standard deviation) of cells expressing IFNβ from three independent ISH experiments. At least 400 cells were blindly counted and scored for each category. (D) L929-RIG-I stable transfectants were transfected with GFP control plasmid. The transfection efficiency was measured by GFP detection using fluorescent microscopy.
(TIF)

Figure S6 **Higher RIG-I and Trim25 protein levels are present in IFNβ-expressing cells.** IFNβ/YFP homozygous MEF cells were infected with SeV for 8 h. Cells were then fixed and intracellularly stained for RIG-I and Trim25. FACS analysis was used to assay the correlation between IFNβ expression (detected by YFP) and RIG-I/Trim25 expression. The top panel shows the expression of RIG-I and Trim25 in MEFs before and after virus infection. The middle panel shows the percentage of IFNβ-expressing cells before and after virus infection. The bottom panel shows the RIG-I/Trim25 expression level in YFP-positive cells in the middle panel, which represent IFNβ-expressing cells. Data shown are representative of at least three independent experiments. Numbers represent relative percentages.
(TIF)

Figure S7 **Over-expression of IRF7 eliminates stochastic IFNα gene expression.** (A) L929-IRF7 stable transfectants were treated with 1 µg/ml tetracycline for 24 h followed by immunocytochemistry using Flag antibody detecting the exogenous Flag-tagged IRF7 expression level. Increased IRF7 expression level was detected in almost every cell. Scale bar, 20 µm. (B) The L929-IRF7 stable transfectant was induced by tetracycline for 24 h, followed by virus infection for 9 h. RNA ISH experiments were carried out to detect IFNα mRNA. (C) Histogram showing the percentage (mean ± standard deviation) of cells expressing IFNα from three independent ISH experiments. At least 400 cells were blindly counted and scored for each category. (D) L929-IRF7 stable transfectants were treated with or without tetracycline for 24 h before SeV infection. Total RNA was extracted and semi-qRT-PCR was carried out to measure the kinetics of transcription of different type I IFN mRNAs.
(TIF)

Figure S8 **IRF7 up-regulates RIG-I-like receptor signaling pathway.** (A) Partial list of genes whose expression in L929-IRF7 cells, as assessed by genome-wide expression profiling, was increased as a result of tetracycline induction to the level of these genes in control cells without tetracycline treatment. The names of known RIG-I-like signaling pathway genes from the KEGG Pathway Database are highlighted in grey. Asterisk indicates that the Ddx58 gene expression profile was undetectable because of the lack of a corresponding probe set on the Affymetrix Mouse Genome 430A_2.0 array chips. The fold increase of Ddx58 gene expression was determined by qRT-PCR. (B) RIG-I-like receptor signaling pathway is the most significantly up-regulated pathway identified from KEGG pathway enrichment analysis (*p* = 3.6E-06) based on L929-IRF7 microarray results. *, *p*<0.05; ***, *p*<0.001. (C) qRT-PCR analysis illustrating the levels of expression of IRF7, RIG-I, Trim25, and MDA5 in L929-IRF7 stable transfectants before and after induction with tetracycline.
(TIF)

Figure S9 **IRF3 and IRF7 are limiting factors in stochastic IFNβ expression induced by poly I∶C transfection.** (A) L929-IRF3, L929-IRF7, or L929-p65 stable transfectants were stimulated with tetracycline for 24 h followed by transient transfection with poly I∶C. 12 h after transfection, cells were fixed followed by RNA ISH to detect IFN-β mRNA. (B) Bar plots representing the percentage (mean ± standard deviation) of cells expressing IFNβ from three independent ISH experiments performed as in (A). At least 400 cells were blindly counted and scored for each category.
(TIF)

Figure S10 **Endogenous variation in the concentrations of components of the RIG-I signaling pathway.** Primary MEF cells were fixed by 4% PFA followed by intracellular staining using appropriate antibodies recognizing different components of the signaling pathway. The intensity of immunofluorescent signal was quantified using ImageJ software. For individual factors, the highest immunofluorescence intensity was set as 1. The *x*-axis shows the relative immunofluorescence intensity of each factor. Each plot represents the immunofluorescence intensity calculated from 150–200 cells.
(TIF)

Figure S11 **Differences in the levels of RIG-I signaling pathway factors between YFP-positive and YFP-negative populations.** (A) qPCR analysis illustrating the expression levels of IFNβ, RIG-I, Trim25, and MDA5 genes in sorted MEF cells at 8, 12, and 24 h.p.i. (B) Western blots showing cytoplasmic distribution of signaling pathway proteins present in FACS-sorted cells at 8, 12, and 24 h.p.i. (C) IFNAR-deficient MEFs were infected by SeV. IFNβ expression was detected by ISH using IFNβ antisense RNA probe.
(TIF)

## Abbreviations

DI: defective interfering
dsRNA: double-stranded RNA
FACS: fluorescence-activated cell sorting
h.p.i.: hours post-infection
HAU: hemagglutination units
IFN: interferon
ISG: interferon-stimulated gene
ISH: in situ hybridization
MEF: mouse embryonic fibroblast
NP: nucleoprotein
poly I∶C: polyriboinosinic polyribocytidylic acid
qPCR: quantitative PCR
qRT-PCR: quantitative reverse transcription PCR
SeV: Sendai virus
