# Supplementary figures and images for: Stochastic Expression of the Interferon-β Gene
Source: PLoS Biol. 2012 Jan 24;10(1):e1001249. doi: 10.1371/journal.pbio.1001249 (PMC3265471; doi:10.1371/journal.pbio.1001249)

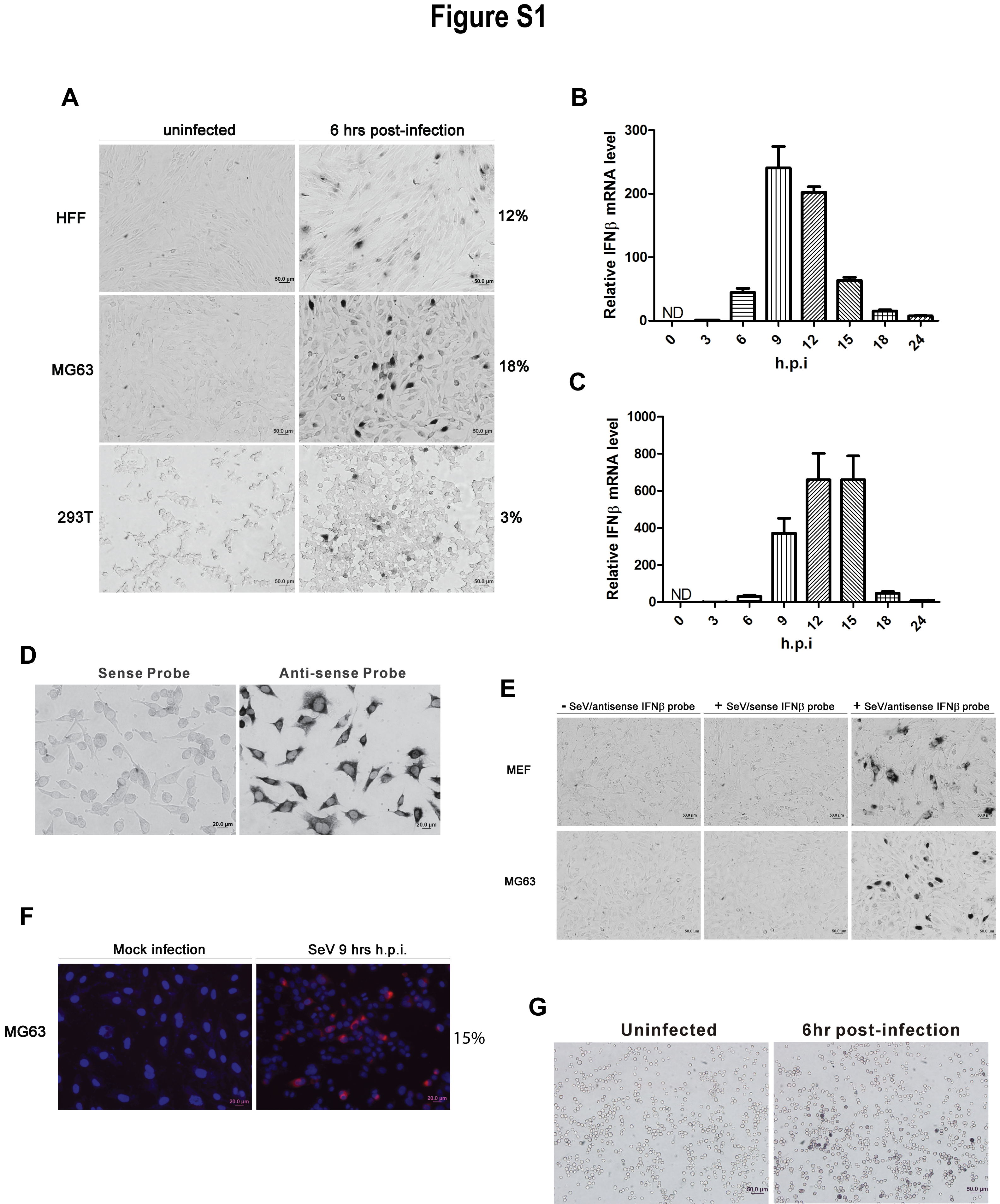

Supplement: Figure S1 — Stochastic expression of IFNβ gene upon virus infection. (A) Stochastic expression of human IFNβ gene in primary foreskin fibroblast cells, MG63 cells, and 293T cells 6 h.p.i., detected by ISH using a digoxygenin-labeled antisense RNA IFNβ probe. Numbers on the right indicate the percentages of IFNβ-expressing cells in each cell type. (B and C) Kinetics of IFNβ expression in primary MEFs (B) or L929 cells (C) assayed by qRT-PCR. IFNβ mRNA can be detected as early as 6 h.p.i., and maximum levels are observed at 9 and 12 h.p.i. in MEFs and L929 cells, respectively. (D) β-actin mRNA in L929 cells was detected by ISH using either digoxygenin-labeled sense or antisense RNA probe. (E) MEF cells or human MG63 cells were infected by SeV for 9 h or 6 h, respectively. ISH was carried out to detect the IFNβ-expressing cells using an IFNβ sense or antisense probe. (F) Human MG63 cells were infected by SeV for 9 h. IFNβ protein was detected by immunocytochemistry using IFNβ antibody. Similar percentages of IFNβ-expressing cell were detected by either ISH or immunocytochemistry. (G) Human IFNα8 mRNA in Namalwa cells was detected by ISH using digoxygenin-labeled probe. (TIF) [file pbio.1001249.s001.tif]

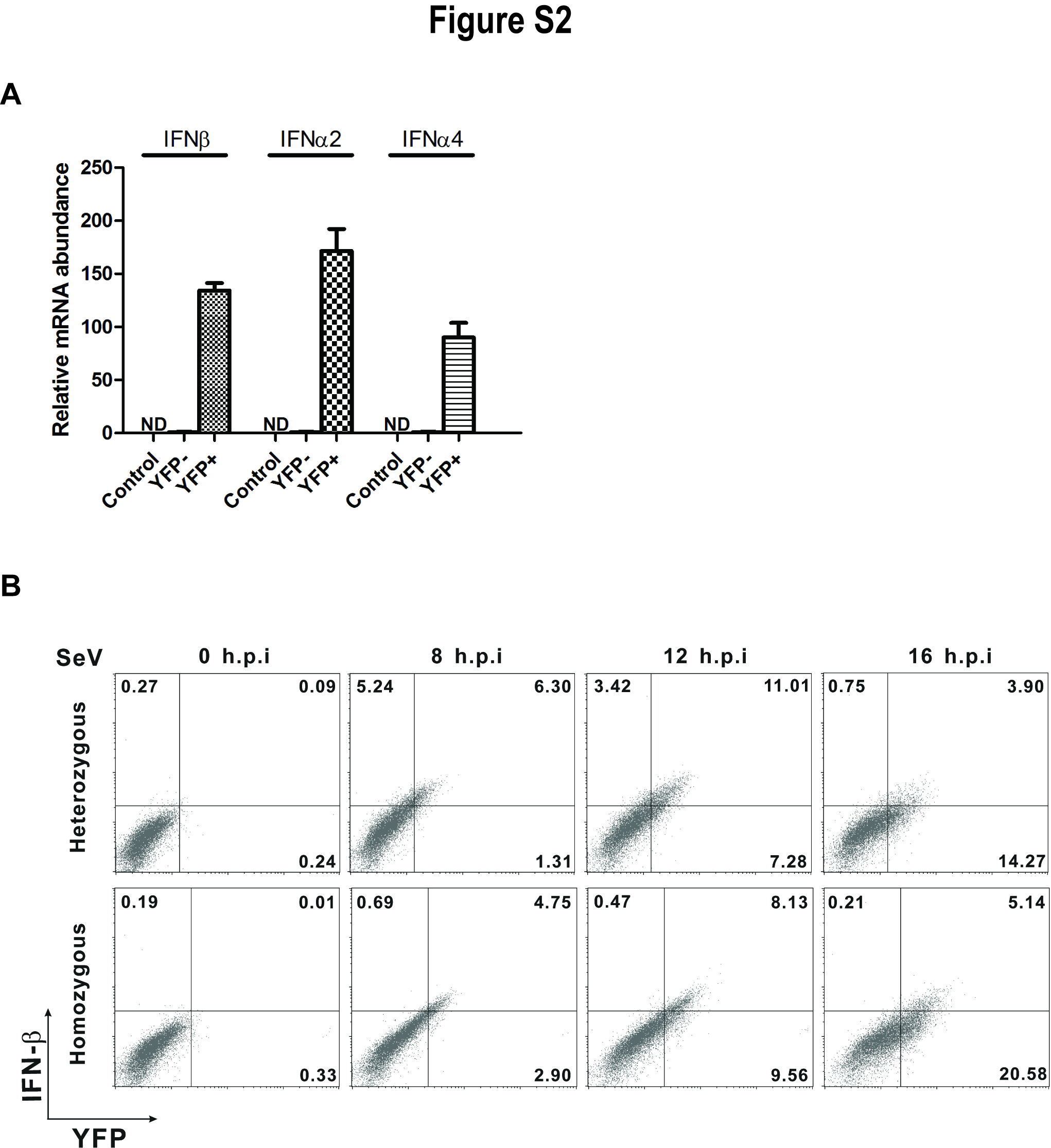

Supplement: Figure S2 — IFN expression in sorted MEFs and allelic expression of IFNβ gene. (A) qRT-PCR analysis illustrating the expression levels of IFN genes in sorted IFNβ/YFP primary MEF cells. (B) IFNβ/YFP heterozygous MEFs (upper panel) and homozygous MEFs (lower panel) were infected by SeV for variable times with the presence of Brefeldin A (BFA)—which inhibits transport of proteins from endoplasmic reticulum to Golgi—in the last 4 h. Cells were fixed and stained for intracellular IFNβ and YFP. If IFNβ gene is monoallelically expressed, heterozygous MEF cells should have similar percentages of IFNβ-positive population (from IFNβ allele) and IFNβ/YFP double-positive population (from IFNβ-IRES-YFP allele). If IFNβ gene is biallelically expressed, all, or at least most, of IFNβ-expressing heterozygous cells should be both IFNβ- and YFP-positive. Heterozygous MEF FACS analysis (upper panel) showed a similar percentage of IFNβ-positive population (upper left panel, 5.24%) and IFNβ/YFP double-positive population (upper right panel, 6.30%) at 8 h.p.i., suggesting that the IFNβ gene expression was predominantly monoallelic before 8 h.p.i. During the time periods 8–12 h.p.i. and 12–16 h.p.i., the majority of IFNβ-expressing cells were IFNβ/YFP double-positive (upper right panel, 11.01%, and upper right panel, 3.90%, respectively), indicating that at late infection, IFNβ gene expression was biallelic. As control, shown in the lower panel, IFNβ-expressing homozygous MEF cells had almost no IFNβ single-positive population at any given time point. Data shown are representative of at least three independent experiments. Numbers represent relative percentages. (TIF) [file pbio.1001249.s002.tif]

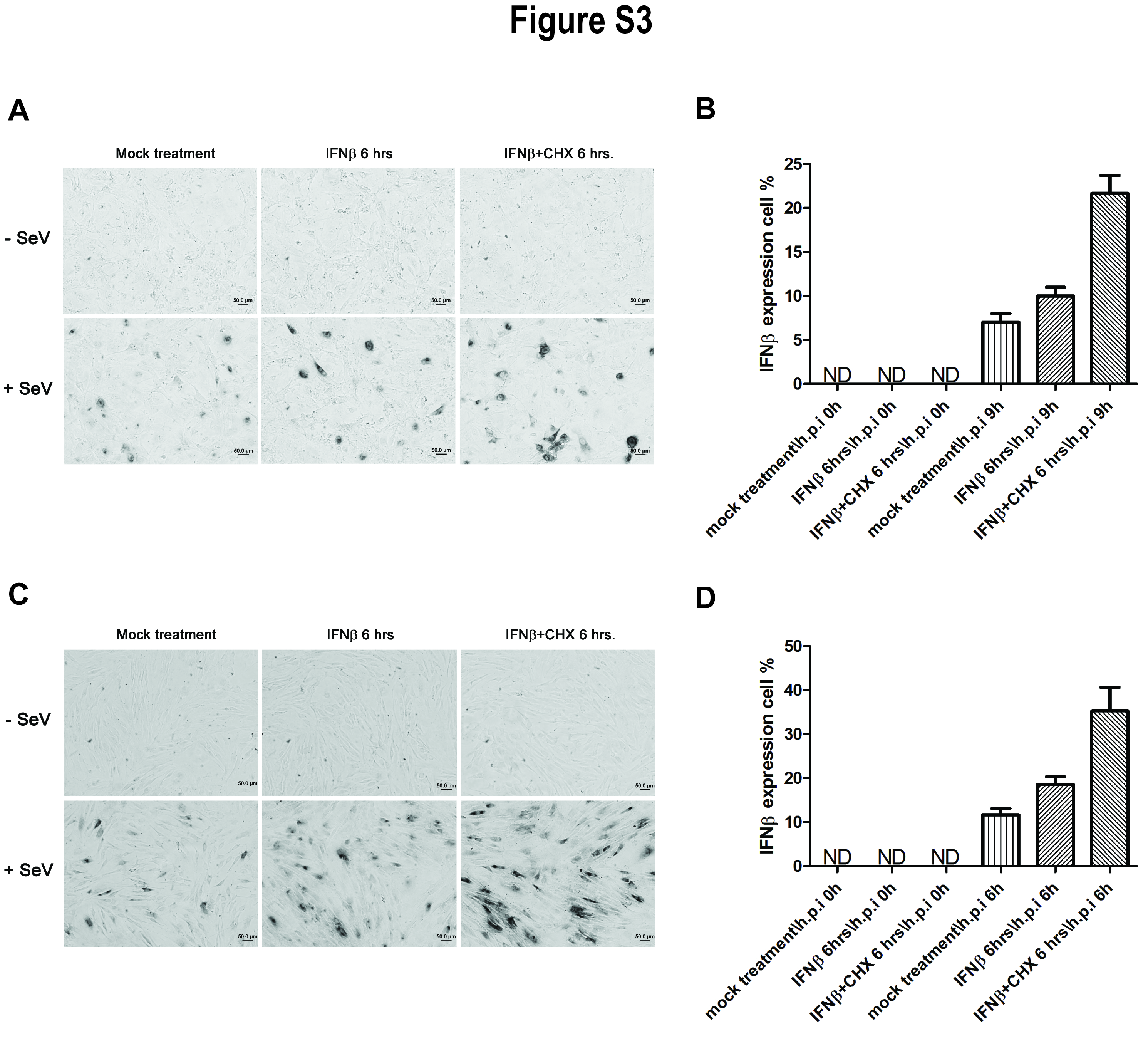

Supplement: Figure S3 — Priming of cells increases the percentage of IFNβ-expressing cells. (A) Primary MEFs were primed with 250 U/ml IFNβ or 250 U/ml IFNβ plus 50 µg/ml cycloheximide (CHX) for 6 h, infected by SeV, and subjected to ISH using digoxygenin-labeled IFNβ RNA probe. (B) Histogram showing the percentage (mean ± standard deviation) of cells expressing IFNβ from three independent ISH experiments as in (A). (C) Human foreskin fibroblasts were primed with 250 U/ml IFNβ or 250 U/ml IFNβ plus 50 µg/ml cycloheximide for 6 h, infected by SeV, and subjected to ISH using digoxygenin-labeled IFNβ RNA probe. (D) Histogram showing the percentage (mean ± standard deviation) of cells expressing IFNβ from three independent ISH experiments as in (C). (TIF) [file pbio.1001249.s003.tif]

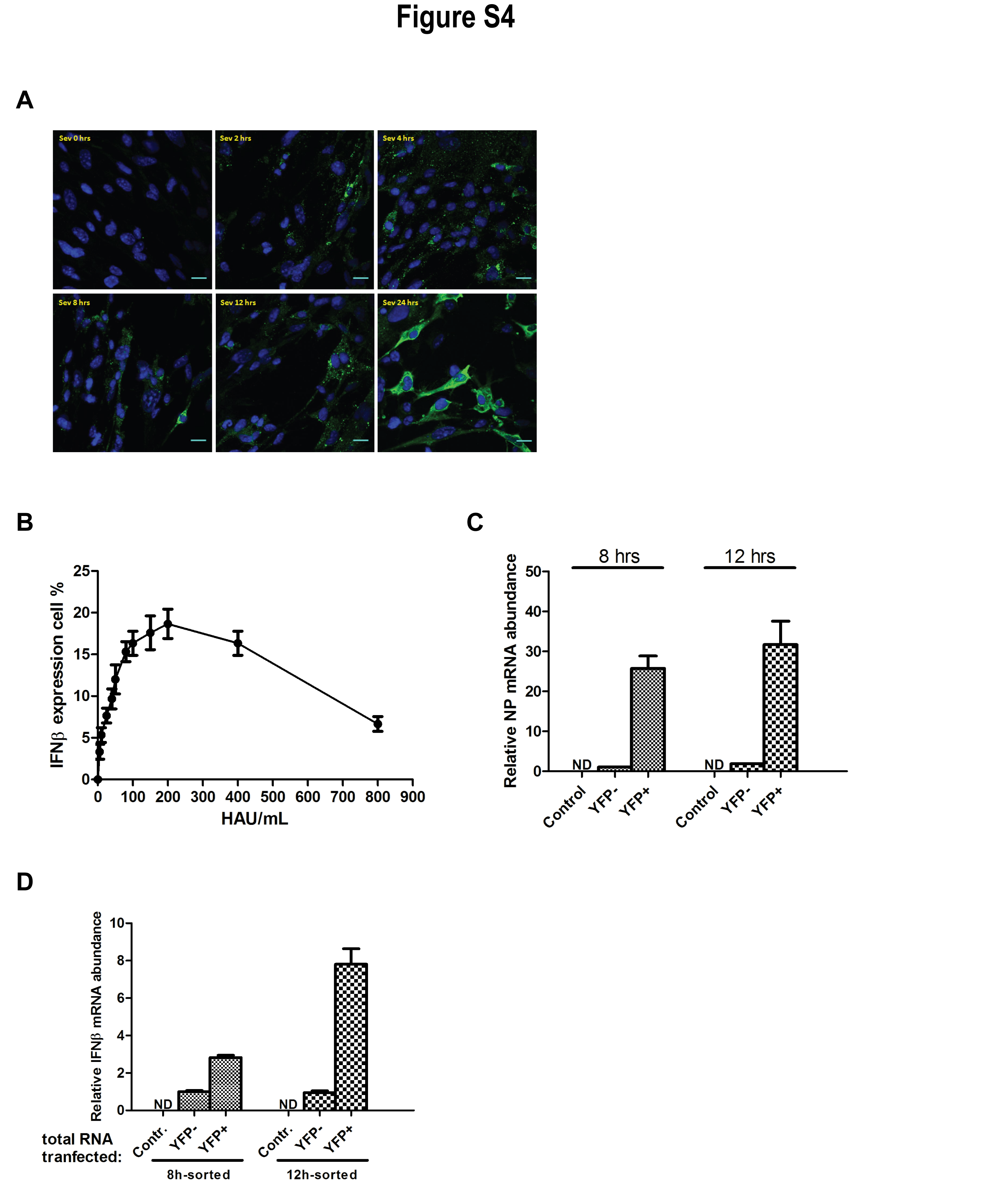

Supplement: Figure S4 — Viral titer is not a limiting factor. (A) MEF cells were infected by SeV. Cells were fixed and stained for SeV using SeV antibody. Blue color shows DAPI staining (nucleus) and green color shows SeV signal. Most, if not all, cells are uniformly exposed to SeV. Scale bar, 20 µm. (B) Percentages (mean ± standard deviation) of IFNβ-producing primary MEF cells infected by increasing amounts of SeV. At least 400 cells were counted and scored blindly for each category. In all of the experiments described in this study, we used 100–200 HAU/ml of SeV as the infecting dose. (C) qRT-PCR analysis illustrating the relative abundance of viral NP mRNA in sorted IFNβ/YFP primary MEF populations 8 or 12 h.p.i. (D) L929 cells were transfected with total RNAs either from IFNβ-producing or IFNβ-nonproducing MEF cells sorted after being virus-infected for 8 h or 12 h. Then total RNAs were extracted from these L929 cells 8 h after transfection, and qRT-PCR experiments were carried out to detect relative abundance of IFNβ mRNA in these transfected cells. (TIF) [file pbio.1001249.s004.tif]

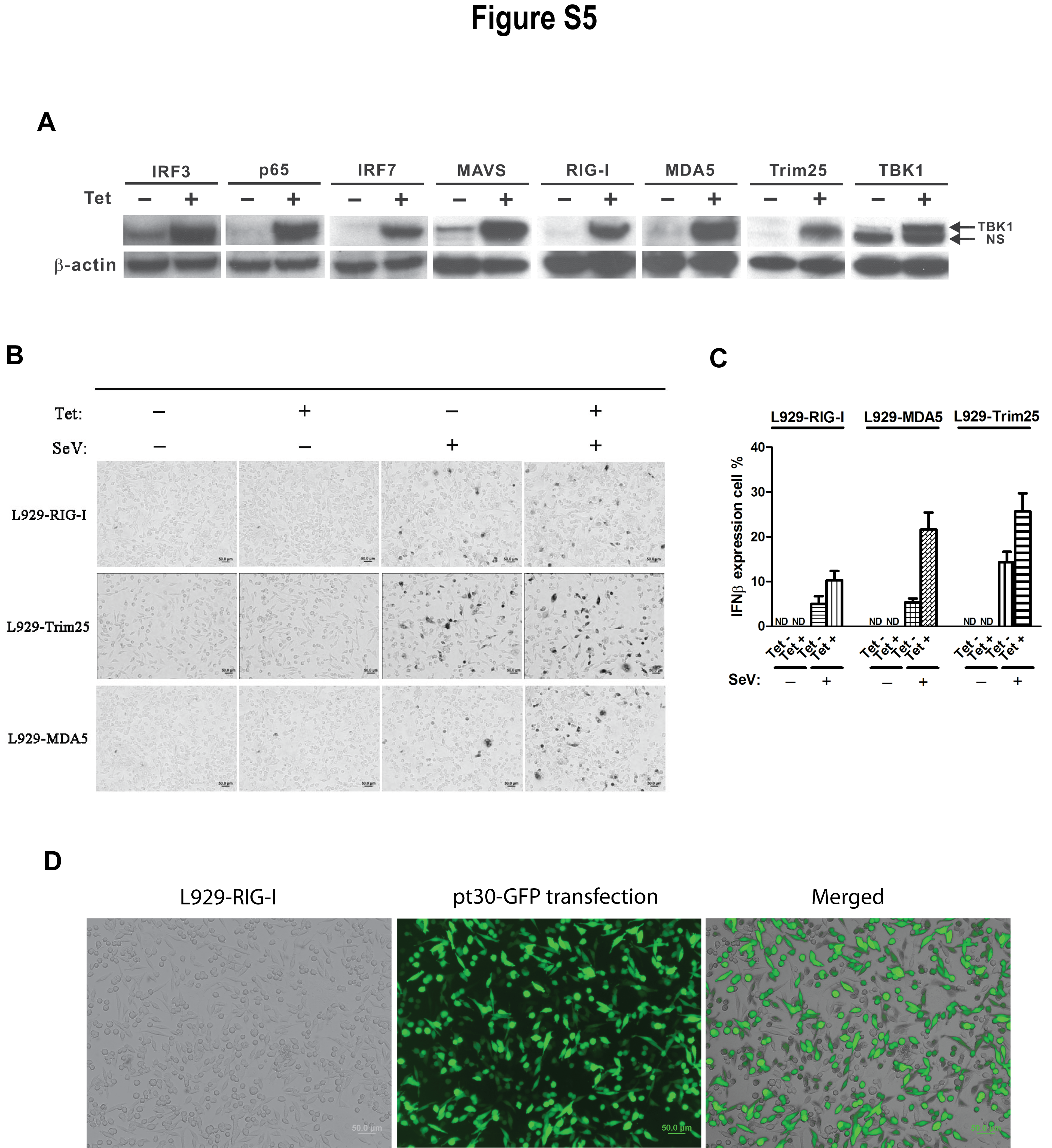

Supplement: Figure S5 — Over-expression of RIG-I, MDA5, or Trim25 increases the percentage of IFNβ-expressing cells. Different tetracycline-inducible L929 stable transfectants were generated. In the absence of tetracycline, expression of exogenous copies of these genes is tightly repressed in the stable transfectants, but upon the addition of 1 µg/ml tetracycline, the stably incorporated genes are expressed at a high level. (A) Western blots showing the tetracycline-inducible expression levels of different proteins. All genes were flag tagged and proteins were detected using Flag antibody. (B) L929-RIG-I, L929-MDA5, and L929-Trim25 stable transfectants were induced by tetracycline for 24 h, followed by virus infection for 9 h. RNA ISH experiments were carried out to detect the IFNβ mRNA. (C) Histogram showing the percentage (mean ± standard deviation) of cells expressing IFNβ from three independent ISH experiments. At least 400 cells were blindly counted and scored for each category. (D) L929-RIG-I stable transfectants were transfected with GFP control plasmid. The transfection efficiency was measured by GFP detection using fluorescent microscopy. (TIF) [file pbio.1001249.s005.tif]

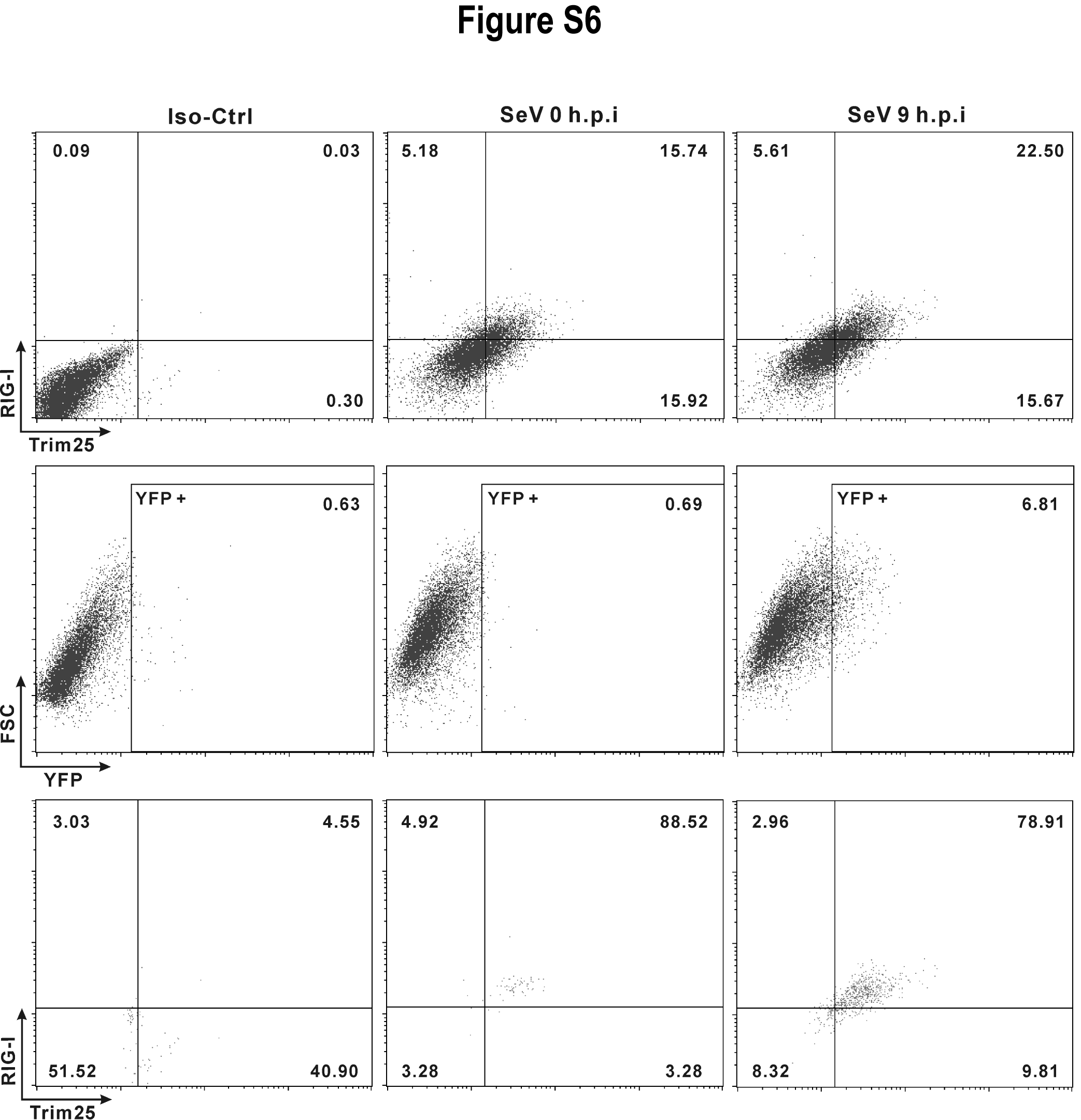

Supplement: Figure S6 — Higher RIG-I and Trim25 protein levels are present in IFNβ-expressing cells. IFNβ/YFP homozygous MEF cells were infected with SeV for 8 h. Cells were then fixed and intracellularly stained for RIG-I and Trim25. FACS analysis was used to assay the correlation between IFNβ expression (detected by YFP) and RIG-I/Trim25 expression. The top panel shows the expression of RIG-I and Trim25 in MEFs before and after virus infection. The middle panel shows the percentage of IFNβ-expressing cells before and after virus infection. The bottom panel shows the RIG-I/Trim25 expression level in YFP-positive cells in the middle panel, which represent IFNβ-expressing cells. Data shown are representative of at least three independent experiments. Numbers represent relative percentages. (TIF) [file pbio.1001249.s006.tif]

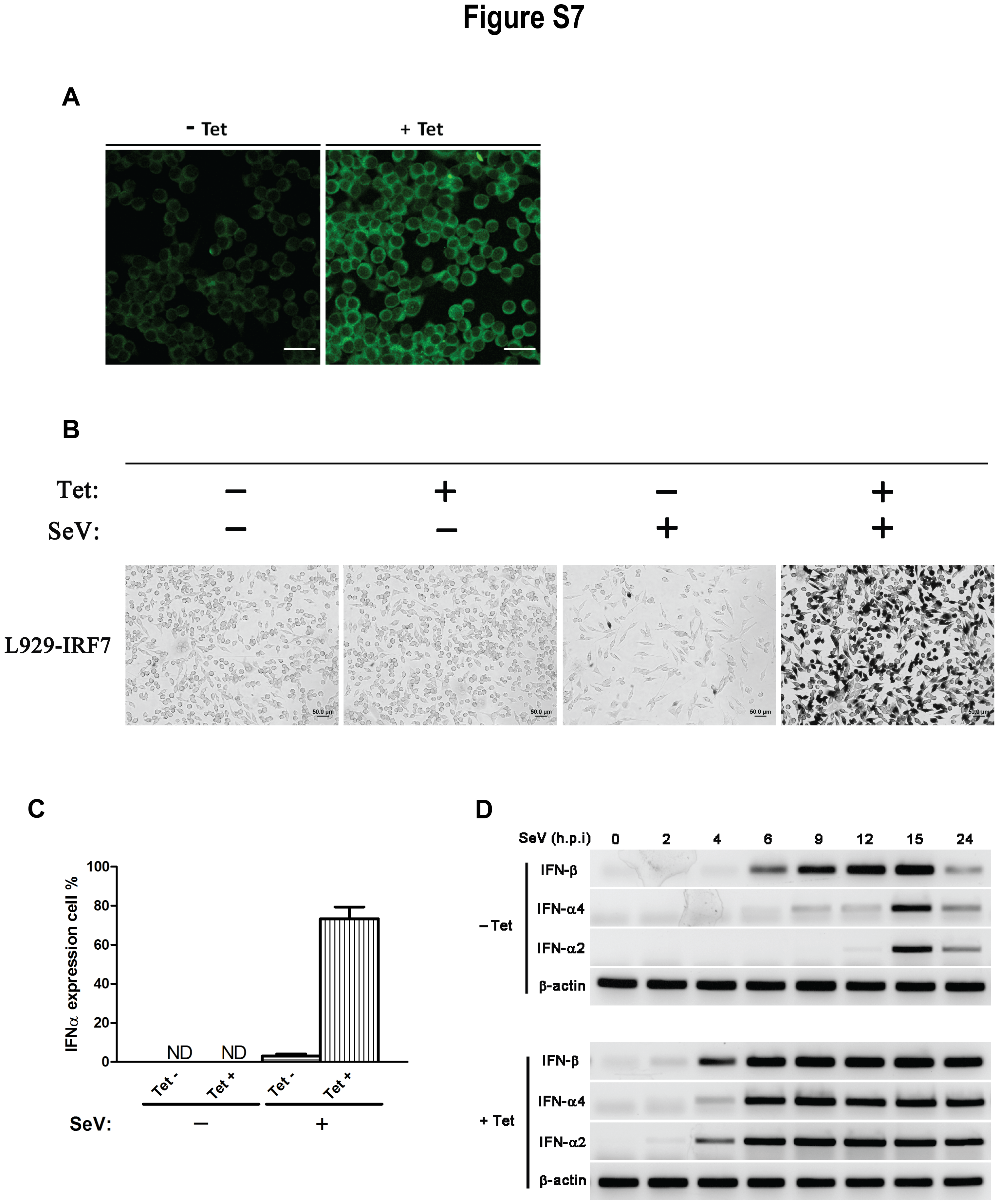

Supplement: Figure S7 — Over-expression of IRF7 eliminates stochastic IFNα gene expression. (A) L929-IRF7 stable transfectants were treated with 1 µg/ml tetracycline for 24 h followed by immunocytochemistry using Flag antibody detecting the exogenous Flag-tagged IRF7 expression level. Increased IRF7 expression level was detected in almost every cell. Scale bar, 20 µm. (B) The L929-IRF7 stable transfectant was induced by tetracycline for 24 h, followed by virus infection for 9 h. RNA ISH experiments were carried out to detect IFNα mRNA. (C) Histogram showing the percentage (mean ± standard deviation) of cells expressing IFNα from three independent ISH experiments. At least 400 cells were blindly counted and scored for each category. (D) L929-IRF7 stable transfectants were treated with or without tetracycline for 24 h before SeV infection. Total RNA was extracted and semi-qRT-PCR was carried out to measure the kinetics of transcription of different type I IFN mRNAs. (TIF) [file pbio.1001249.s007.tif]

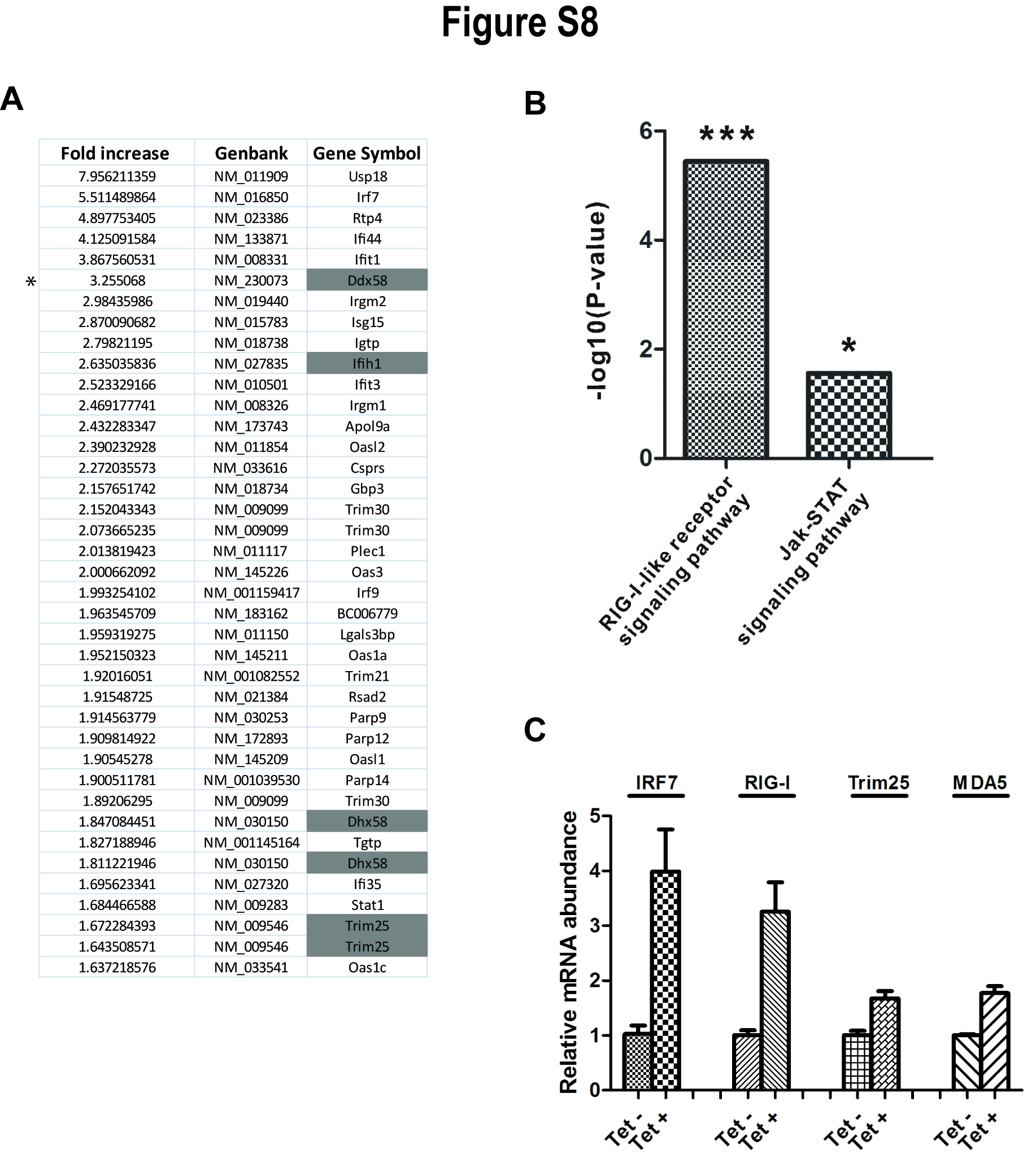

Supplement: Figure S8 — IRF7 up-regulates RIG-I-like receptor signaling pathway. (A) Partial list of genes whose expression in L929-IRF7 cells, as assessed by genome-wide expression profiling, was increased as a result of tetracycline induction to the level of these genes in control cells without tetracycline treatment. The names of known RIG-I-like signaling pathway genes from the KEGG Pathway Database are highlighted in grey. Asterisk indicates that the Ddx58 gene expression profile was undetectable because of the lack of a corresponding probe set on the Affymetrix Mouse Genome 430A_2.0 array chips. The fold increase of Ddx58 gene expression was determined by qRT-PCR. (B) RIG-I-like receptor signaling pathway is the most significantly up-regulated pathway identified from KEGG pathway enrichment analysis (p = 3.6E-06) based on L929-IRF7 microarray results. *, p<0.05; ***, p<0.001. (C) qRT-PCR analysis illustrating the levels of expression of IRF7, RIG-I, Trim25, and MDA5 in L929-IRF7 stable transfectants before and after induction with tetracycline. (TIF) [file pbio.1001249.s008.tif]

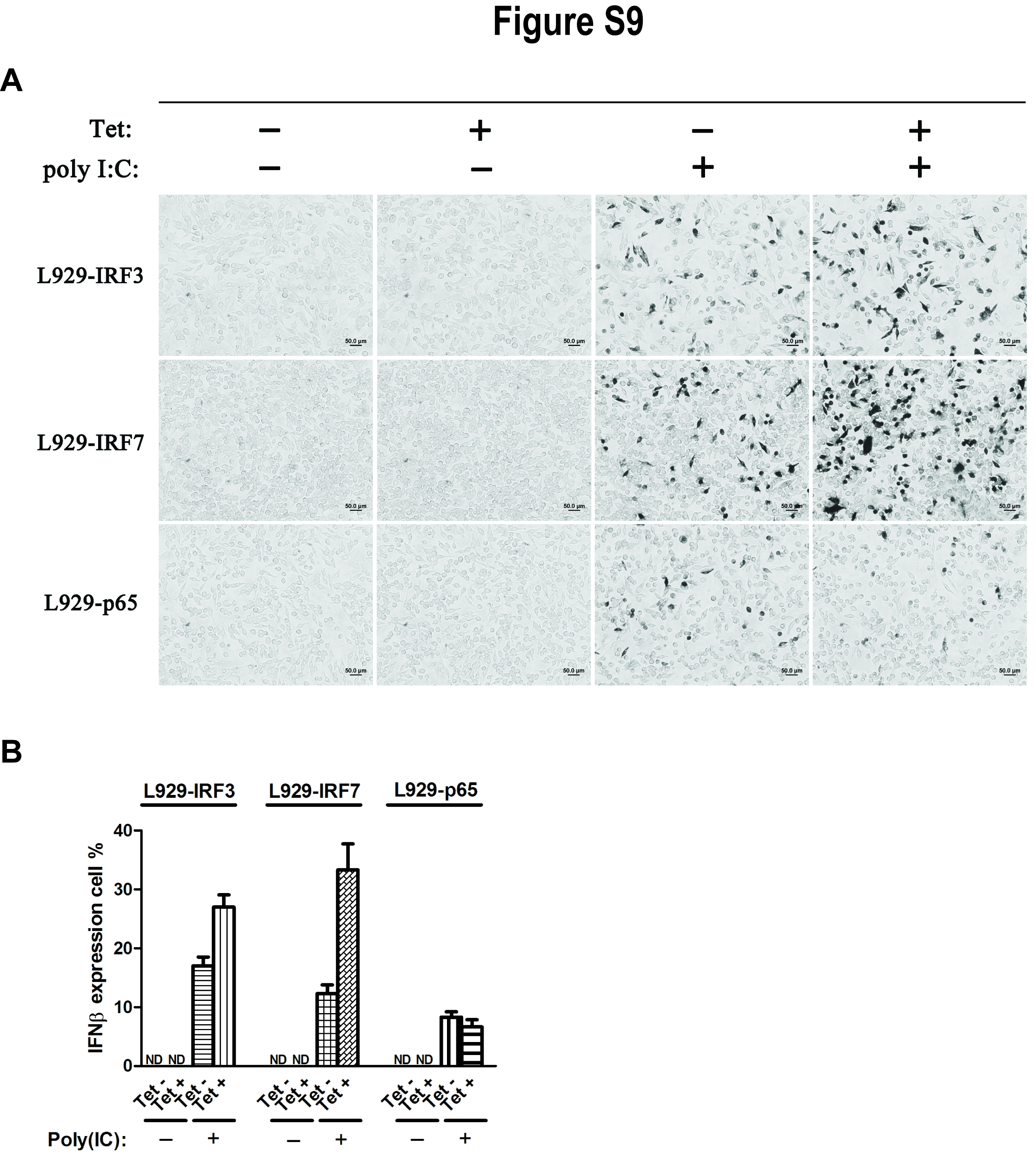

Supplement: Figure S9 — IRF3 and IRF7 are limiting factors in stochastic IFNβ expression induced by poly I∶C transfection. (A) L929-IRF3, L929-IRF7, or L929-p65 stable transfectants were stimulated with tetracycline for 24 h followed by transient transfection with poly I∶C. 12 h after transfection, cells were fixed followed by RNA ISH to detect IFN-β mRNA. (B) Bar plots representing the percentage (mean ± standard deviation) of cells expressing IFNβ from three independent ISH experiments performed as in (A). At least 400 cells were blindly counted and scored for each category. (TIF) [file pbio.1001249.s009.tif]

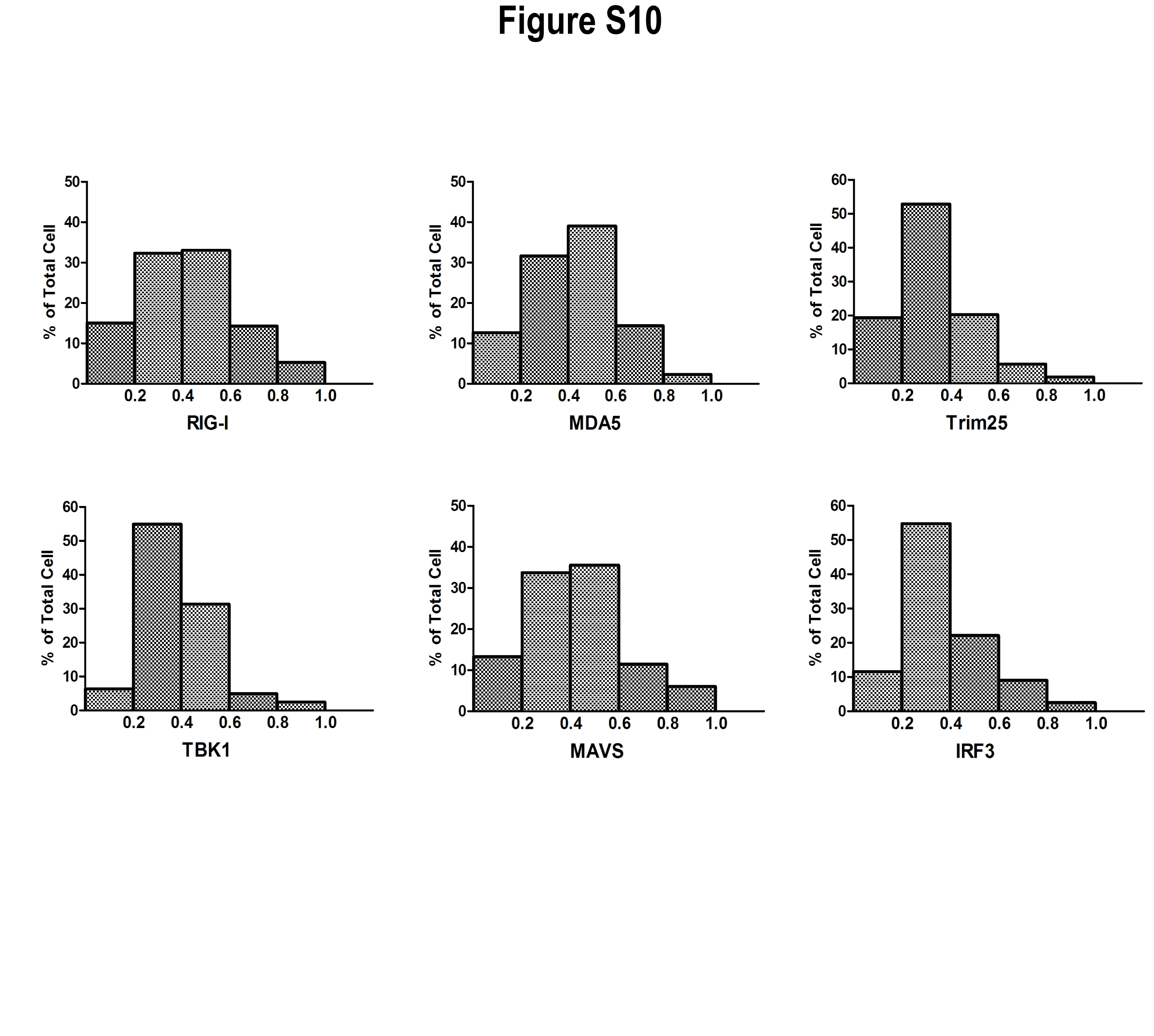

Supplement: Figure S10 — Endogenous variation in the concentrations of components of the RIG-I signaling pathway. Primary MEF cells were fixed by 4% PFA followed by intracellular staining using appropriate antibodies recognizing different components of the signaling pathway. The intensity of immunofluorescent signal was quantified using ImageJ software. For individual factors, the highest immunofluorescence intensity was set as 1. The x-axis shows the relative immunofluorescence intensity of each factor. Each plot represents the immunofluorescence intensity calculated from 150–200 cells. (TIF) [file pbio.1001249.s010.tif]

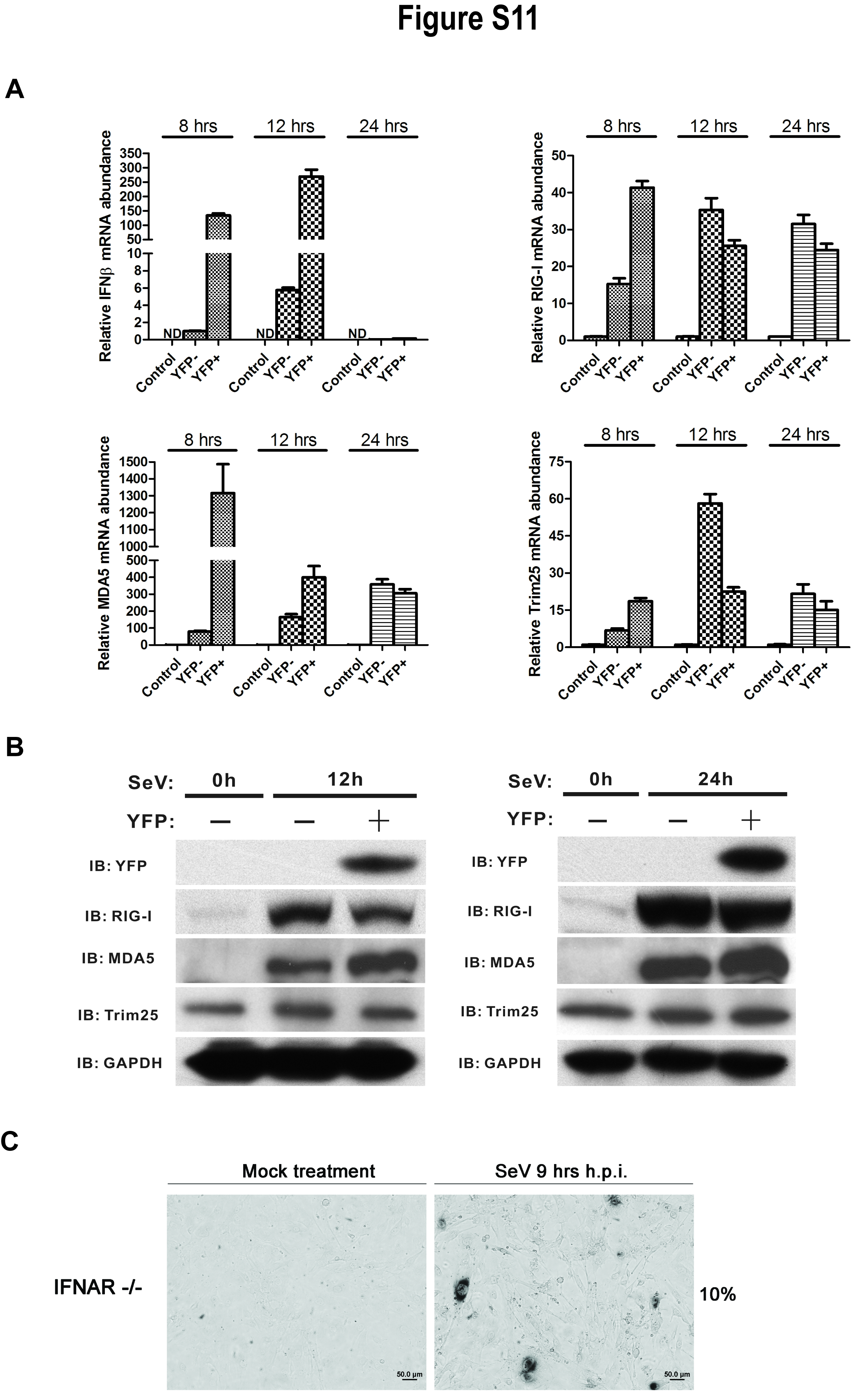

Supplement: Figure S11 — Differences in the levels of RIG-I signaling pathway factors between YFP-positive and YFP-negative populations. (A) qPCR analysis illustrating the expression levels of IFNβ, RIG-I, Trim25, and MDA5 genes in sorted MEF cells at 8, 12, and 24 h.p.i. (B) Western blots showing cytoplasmic distribution of signaling pathway proteins present in FACS-sorted cells at 8, 12, and 24 h.p.i. (C) IFNAR-deficient MEFs were infected by SeV. IFNβ expression was detected by ISH using IFNβ antisense RNA probe. (TIF) [file pbio.1001249.s011.tif]
